# Conservation Genetic Assessment of Savannah Elephants (*Loxodonta africana*) in the Greater Kruger Biosphere, South Africa

**DOI:** 10.3390/genes10100779

**Published:** 2019-10-05

**Authors:** Teresa L. Santos, Carlos Fernandes, Michelle D. Henley, Deborah A. Dawson, Hannah S. Mumby

**Affiliations:** 1 Bull Elephant Network Project, Conservation Science Group, David Attenborough Building, Pembroke St, Cambridge CB2 3QY, UK; 2 NERC Biomolecular Analysis Facility, Department of Animal and Plant Sciences, University of Sheffield, Western Bank, Sheffield, South Yorkshire S10 2TN, UK; 3 cE3c - Centre for Ecology, Evolution and Environmental Changes, Department of Animal Biology, Faculty of Sciences, University of Lisbon, 1749-016 Lisbon, Portugal; 4 Applied Behavioural Ecology and Ecosystem Research Unit, University of South Africa, Florida Campus, Private Bag X6, Florida 1710, Johannesburg, South Africa; 5 Elephants Alive, P.O. Box 960, Hoedspruit 1380, South Africa; 6 Centre for African Ecology, School of Animal, Plant and Environmental Sciences, University of Witwatersrand, 1 Jan Smuts Avenue, Braamfontein, Johannesburg 2000, South Africa; 7 Wissenschaftskolleg zu Berlin, Wallotstraße 19, 14193 Berlin, Germany; 8 School of Biological Sciences and Department of Politics and Public Administration, University of Hong Kong, Hong Kong, China

**Keywords:** *Loxodonta africana*, Greater Kruger National Park (GKNP), conservation genetics, genetic structure, effective population size, demographic history

## Abstract

Savannah elephant populations have been severely reduced and fragmented throughout its remaining range. In general, however, there is limited information regarding their genetic status, which is essential knowledge for conservation. We investigated patterns of genetic variation in savannah elephants from the Greater Kruger Biosphere, with a focus on those in previously unstudied nature reserves adjacent to Kruger National Park, using dung samples from 294 individuals and 18 microsatellites. The results of genetic structure analyses using several different methods of ordination and Bayesian clustering strongly suggest that elephants throughout the Greater Kruger National Park (GKNP) constitute a single population. No evidence of a recent genetic bottleneck was detected using three moment-based approaches and two coalescent likelihood methods. The apparent absence of a recent genetic bottleneck associated with the known early 1900s demographic bottleneck may result from a combination of rapid post-bottleneck population growth, immigration and long generation time. Point estimates of contemporary effective population size (N_e_) for the GKNP were ~ 500–700, that is, at the low end of the range of N_e_ values that have been proposed for maintaining evolutionary potential and the current ratio of N_e_ to census population size (N_c_) may be quite low (<0.1). This study illustrates the difficulties in assessing the impacts on N_e_ in populations that have suffered demographic crashes but have recovered rapidly and received gene flow, particularly in species with long generation times in which genetic time lags are longer. This work provides a starting point and baseline information for genetic monitoring of the GKNP elephants.

## 1. Introduction

Conservation threats, such as illegal hunting, habitat fragmentation and confinement to small isolated areas, contribute to the decline of animal populations and can lead to their extinction [1,2,3]. The impacts on wildlife can take many forms, for example on reproductive success [4], social interactions [5] and dispersal [6]. Subtler but overarching are the effects on genetic diversity and structure. For instance, a decrease in population size is often accompanied by an increase in inbreeding and a reduction in genetic variation [7]. Diminished genetic variability and genetic erosion have been associated with lowered population fitness, limited ability to adapt to environmental changes and a higher risk of population extinction [8,9]. Anthropogenic barriers and landscape fragmentation can impede gene flow, disrupt long-standing spatial genetic patterns and impose strong structuring of the genetic diversity of populations and species, even highly mobile ones [10]. Thus, human structures and activities, besides being potential threats to wildlife, may also be important drivers of contemporary microevolution [11]. 

Complex, cryptic and sensitive fine-scale population genetic structure is often observed in social animals [12], including elephants [13]. The African savannah elephant (*Loxodonta africana*) is a charismatic flagship species that continues to decline in numbers due to poaching and habitat destruction [14,15], being listed as Vulnerable in the IUCN Red List of Threatened Species [16]. Elephants are unusual behaviourally because, unlike most other social mammals, they live in fluid fission-fusion societies with a hierarchical structure, where males never form permanent breeding associations with female groups and reproduce with females from across the entire population [17]. Elephant social behaviour shapes and is shaped by genetic structure and thus understanding these relationships and how humans are impacting them, is crucial for the conservation of elephants [17].

Over the last 200 years, African elephant numbers decreased from more than 20 million to ~1.3 million in the late 1970s [18]. Intense poaching for ivory during that decade and the next [19] led to approximately a further halving of continental elephant numbers at the end of the 1980s [18]. A new and growing wave of poaching in the last two decades has caused an estimated annual decrease of 8% (~30,000) of the less than 400,000 remaining elephants in Africa [14]. Poaching is not the only threat to elephant populations, since hunting pressure and habitat encroachment by humans are increasingly confining elephants to protected areas that account for only 16% of their distributional range and have limited carrying capacity [20]. Moreover, the rapid human population growth and the associated expansion of agriculture and livestock production in elephant range countries have increased human-elephant conflict [2]. Today, about 70% of savannah elephants occur in Southern Africa and the largest populations, because they have a higher probability of long-term persistence, are arguably the most critical to the future survival of the species [14]. Previous studies have shown that savannah elephants in eastern and southern Africa are significantly differentiated at the mtDNA level [21], more moderately so in microsatellites [22] and indistinguishable at some autosomal and sex-linked nuclear genes [23,24]. In South Africa, during the eighteenth and nineteenth centuries, human encroachment, hunting for ivory and shooting for crop protection led to a nationwide low of 120 animals in 1920 [25]. Subsequent protection and management allowed an increase in the species’ numbers, with most of the recovery occurring in the Kruger National Park (KNP), with growth by recruitment boosted by immigration from neighbouring Mozambique [25], so that the KNP population (~17,000) [14] currently accounts for more than 60% of South Africa’s elephants [26].

Among the expected consequences of the continued decline and fragmentation of elephant populations are reduced genetic variation and increased inbreeding, which may lead to inbreeding depression and decreased adaptability and evolutionary potential [27] and greater inter-population differentiation [28]. Effective conservation management of the remaining populations should therefore take these genetic issues into account [29]. Here, we conducted a conservation genetic assessment of savannah elephants in the Greater Kruger Biosphere in South Africa, with a focus on those in hitherto unstudied nature reserves adjacent to the KNP, which were separated by fences for about three decades but are now unfenced toward the KNP. Specifically, using 18 *L. africana* microsatellite markers, we genotyped dung samples from 294 individuals and investigated genetic diversity and structure, effective population size and demographic history. Microsatellites have been successfully used in many conservation genetic assessments (e.g., Reference [28]). The suitability of mitochondrial DNA (mtDNA) for the study of overall current population structure in savannah elephants has been called into question due to their female philopatry and male-mediated gene flow [30]. 

## 2. Materials and Methods 

### 2.1. Study Area and Sample Collection

The Associated Private Nature Reserves (APNR) in South Africa are a series of privately-owned nature reserves (Klaserie, Timbavati, Balule and Umbabat), which cover ca. 1800 km^2^. They are continuous with the western boundary of the KNP, which in turn is contiguous with Mozambique’s Limpopo National Park (LNP) to the east. Since the early 1990s no fences separate the APNR from the KNP, while sections of fence were dropped with LNP in the early 2000s, ending almost 30 years of elephant barrier to and from Mozambique [31]. Field-based research has been conducted since 1996 and officially since 2003, on the elephants in this area by the South African non-profit organisation Elephants Alive. Elephants Alive has identified to date over 1500 individual elephants [32]. 

**Figure 1:**
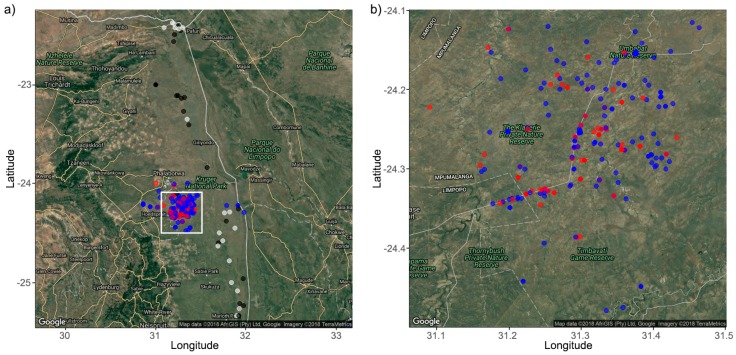
Location of samples used in this study. Blue dots are males from the Associated Private Nature Reserves (APNR); black dots, males from the Kruger National Park (KNP); red dots, females from the APNR; and white dots, females from the KNP. (**a**) Map showing the locations of all samples; (**b**) Enlarged map of the area delimited by the white square in a), where most of the APNR samples were collected.

A total of 360 savannah elephant dung samples were collected in the APNR between late October 2015 and mid-April 2017. In addition, 46 samples, collected in the KNP, were kindly provided by Samuel Wasser’s team at the University of Washington (Figure 1). These samples were already diluted to an average of 20 ng/μL DNA in ‘Low TE’ buffer (10 mM Tris–HCl, 0.1 mM EDTA). From the APNR samples, 45 were preserved in absolute ethanol and the remaining ones in a saturated salt solution (circa 36 g of iodised table salt per 100 mL of mineral H_2_O) and for 29 there was a replicate from the same dung preserved in both ethanol and salt solution. All samples were freshly collected from elephants that were observed defecating. GPS coordinates were obtained for every sample upon collection. Each individual’s age was estimated [33]. IDs were given to every individual based on ear and tusk characteristics observed in photographic evidence [34]. 

### 2.2. Laboratory Procedures and Microsatellite Quality Control

DNA was extracted using the QIAamp Fast DNA Stool Mini Kit (QIAGEN). Samples were genotyped for 18 species-specific autosomal microsatellite loci [35,36,37] and three sex-typing markers [38]. All samples were initially genotyped twice for each locus to verify the genotypes. We performed additional replicates until matching heterozygotes were scored at least twice and matching homozygotes at least three times [39,40].

Consensus genotypes were built on Gimlet 1.3.3 [41], which also provided estimates of allelic dropout, false alleles and five other specific types of genotyping errors [41,42]. Unbiased P_ID_ and P_ID-SIB_ were calculated to quantify the power of the microsatellite loci to differentiate individuals [43]. Resampled individuals and possible mismatches due to genotyping errors were identified in Cervus 3.0.7 [44]. Observed and expected distributions of genotypic differences between samples were computed in MM-Dist [45]. Large allele dropout and null alleles were assessed using Micro-Checker 2.2.3 [46]. Hardy-Weinberg and linkage equilibria among loci were tested in GENEPOP 4.7.0 [47] and significance levels (α = 0.05) were adjusted using the sequential Holm-Bonferroni procedure [48] in an Excel calculator [49]. Combining tests provides greater confidence in null allele detection [50]. Thus, in addition to Micro-Checker and the method of Van Oosterhout et al. [46], presence and frequency of null alleles were further assessed using an iterative estimator [51] implemented in Cervus, a maximum-likelihood (ML) estimator [52] implemented in ML-NullFreq and both an individual inbreeding model (IIM; Bayesian) and a population inbreeding model (PIM; ML) approaches [53] available in Inest 2.2. Further details on laboratory procedures and microsatellite quality control can be found in the Supplementary Information.

### 2.3. Genetic Diversity and Structure

Genetic diversity was quantified using standard summary statistics calculated for each locus and averaged over all loci. Number of alleles and observed and expected heterozygosities were computed using Cervus. Allelic richness and private allelic richness for APNR and KNP were determined with HP-Rare 1.1 [54]. The inbreeding coefficient (F_IS_) was estimated using Genetix 4.05.2 [55] and the significance was tested based on 10000 permutations of alleles among individuals. Apart from our interest in the elephants in the APNR and their comparison with those in the KNP, estimates of genetic diversity were quantified separately for them also because they, despite the current absence of fences between the two areas, should not be assumed to be genetically homogeneous for several reasons. First, after the demographic collapse of the KNP elephants at the turn of the 19th century, their recovery and recolonization of the area corresponding to the present-day Greater Kruger National Park (GKNP) was gradual, having been earlier and more intense in the centre and north of the park and later and more protracted to the south [56]. This, coupled with spatially differential immigration and local founder effects [57], may have generated genetic structure throughout the GKNP. Concordantly, substantial mtDNA differentiation has been observed between samples from the north and south of the KNP, which suggests that elephant recovery and recolonization of the Kruger area may have had the contribution of founders and immigrants from different nearby geographic sources [30]. Second, for about three decades (early 1960s to the early 1990s) the KNP and the private land now part of the APNR were separated by fences that should have acted as semi-permeable barriers to elephant movements [58,59,60]. Third, in elephant populations previously restricted by fences, resident individuals, particularly family groups but also adult males, can act cautiously in exploring new areas, even if unoccupied and thus may expand their home ranges very slightly and slowly into a new area made available after boundary fence removal [61]. Fourth, savannah elephants in Southern Africa prefer habitats close to water and with high proportion of vegetation of different types and may move little within seasons if these essential resources remain locally abundant [62]. Given the above four arguments, in combination with the long generation time of elephants relative to the time since the fences between the APNR and the KNP were removed, we considered that a lack of genetic differentiation between the two areas should not be assumed at the outset.

We tested for fine-scale genetic structure using multilocus spatial autocorrelation (SAC) analyses of adults in GenAlEx 6.502 [63,64]. The autocorrelation coefficient *r* [65], which provides a measure of the genetic similarity between pairs of individuals falling within a given distance class, was calculated for a specified number of distance classes. The coefficient, which is closely related to Moran’s *I* [66], is bounded by −1 and +1 and has a mean of zero when there is no autocorrelation [65]. The detection of spatial autocorrelation is influenced by the size of distance classes and by the number of samples per distance class [67]. Therefore, analyses were performed using either even distance classes or even samples classes. In the second option, we tried to maximise the number of classes while keeping a minimum of 100 pairwise comparisons per distance class to ensure adequate statistical power [68]. SAC analyses are based on a single location per individual; for individuals observed multiple times, we used the mean of locations (e.g., Reference [69]). 9999 random permutations of individuals among locations were used to generate a 95% confidence envelope under the assumption of no spatial structure. 95% CIs around estimates of *r* were obtained by 9999 bootstraps from within the set of pairwise comparisons for each distance class. We accepted significance only when both *r* exceeded the 95% CI around the null hypothesis of *r* = 0 and the 95% bootstrap CI about *r* did not contain zero [67]. Correlograms were produced by plotting *r* as a function of distance class and their overall significance was obtained using a heterogeneity test [70]. The extent of positive spatial genetic structure was not estimated from the first x-intercept of the correlogram because its value depends on the chosen distance class size. Instead, it was approximated by the distance class size at which the estimate of *r* was no longer significant [67]. Elephants exhibit female philopatry while males disperse from their natal social group at maturity. To examine sex differences in fine-scale spatial genetic structure, we also performed SAC analyses separately for females and males. The statistical significance of differences in spatial genetic structure patterns between adults of each sex was assessed using two heterogeneity tests at the distance class and whole correlogram levels [70]. We focused on the first distance class because, in the case of sex-biased dispersal, any differences in spatial autocorrelation between the sexes are expected to be most apparent in this class [71]. As the APNR elephant samples were collected over a period corresponding to approximately three consecutive seasons (wet-dry-wet), the fact that elephants can seasonally change the size and location of their home ranges could be a confounding factor in the SAC analyses. 65% of the APNR adult samples (71% of the females and 62% of the males) were from the second (dry) season; for this reason, it was only possible to conduct separate analyses for this season, whereas for the other two seasons the sample size was too small (less than 100 pairwise comparisons per distance class).

We tested for isolation by distance (IBD) separately for the APNR and KNP and overall using Mantel tests [72] for correlation between pairwise genetic and geographic distances among adult individuals [73]. The Mantel tests were performed in GenAlEx and analyses were carried out using both raw and log-transformed geographic distances. Significance testing was performed using 9999 permutations. As genetic distances, we used both the Rousset’s distance [74] and Nason’s kinship coefficient [75] as calculated in SPAGeDi 1.5 [76]. We also analysed patterns of IBD for each sex; these analyses were not carried out for the KNP alone because of small sample sizes. Tests of IBD overall and for each sex were also performed using only the APNR adult samples from the second (dry) season.

To assess and visualize the genetic affinities among individuals, multivariate representations were obtained by subjecting the data to a factorial correspondence analysis (FCA) in Genetix and a principal coordinates analysis (PCoA) of the standardized covariance of a codominant genotypic distance [65], as implemented in GenAlEx. Analyses were run for all individuals and for females and males only.

We investigated the presence of genetic structure across the APNR and KNP using Bayesian clustering methods implemented in Structure 2.3.4 [77], Geneland 4.0.8 [78] and Tess 2.3.1 [79] to determine the most likely number of genetic clusters (*K*). *K* was estimated for all individuals and for each sex separately. Structure is the most widely used and thoroughly tested Bayesian clustering software (e.g., References [80,81]) but is aspatial, while Geneland and Tess provide spatially explicit models that use the geographic coordinates of individuals as prior information and therefore can be more powerful at detecting subtle population structure [82]. The analyses with the different methods used, whenever possible, similar run parameters, in order to make the results more comparable. We tested values of *K* from 1 to 8 and the settings for each of the algorithms were as in Basto et al. [83], with the following few exceptions. In Geneland, we conducted 15 independent runs for each *K* value tested, the coordinate uncertainty was set to 0.01, the option to account for the presence of null alleles was selected and the correlated allele frequencies model was run using either the default medium-sized differentiation prior *β* (2,20), an uninformative differentiation prior *β* (1,1) or a low differentiation prior *β* (1,100). It is important to conduct analyses with different *β* priors on the drift parameters because they affect the inference of *K* and individual ancestry using the correlated frequency model [84,85]. In Structure, each run consisted of 500,000 post-burn-in iterations and to visualize results for the most likely *K* the output from Structure Harvester 0.6.94 [86] was processed in CLUMPP 1.1.2 [87] and plotted with distruct 1.1 [88]. If the most supported *K* was 1, the results for *K* = 2 were also examined. We also carried out additional confirmatory analyses, using Tess and the uncorrelated model in Geneland, in which the only APNR samples included were those from the second (dry) season.

Finally, we tested for genetic differentiation between APNR and KNP using an exact allelic G-test [89], without assuming random mating within samples, in Fstat 2.9.3.2 [90] with 20,000 randomizations to assess significance. We also calculated Fst [91], G’’st [92] and Jost’s Dest [93], in Genodive 2.0b27 [94]; significance was tested using 20,000 permutations and 95% CIs were obtained by bootstrapping across loci. Analyses were further conducted only for adults, both overall and by sex. Sex-specific Fst estimates for adults were compared using a permutation test that has been shown to be powerful for detecting sex-biased dispersal [95]; the analysis was performed in Fstat and one-sided p-values for the hypothesis that Fst is significantly higher in females, the philopatric sex, were based on 10,000 randomizations.

### 2.4. Effective Population Size (N_e_) and Demographic History

Effective population size (N_e_) [96] was estimated with the linkage disequilibrium (LD) method of Waples [97] in NeEstimator 2.1 [98], which implements an improved method to account for missing data [99]. We assumed random mating and, as recommended by Waples and Do [100] for sample sizes >25, excluded alleles with frequencies less than 0.02. 95% CIs were calculated by jack-knifing over individuals [101]. LD estimates of local N_e_ are robust to equilibrium immigration rates up to 10% [102,103] and to skewed sex ratio and non-random variance in reproductive success [97]. The LD estimator can accommodate allelic dropout rates up to 0.05 [104]. Moreover, results in Sved et al. [105] indicate that the LD method appears to work well in the presence of null alleles and null allele simulations in Reference [104] suggest that frequencies of null alleles per locus up to 0.1 do not significantly bias N_e_ estimates obtained from the LD method. To compare results and considering the potential effects of population subdivision [106,107], N_e_ was estimated for the APNR and APNR + KNP. N_e_ estimates were based only on adult individuals, as this is preferable to whole-population samples when applying the LD method to iteroparous species [108,109]. Using only samples of adults can also often result in downwardly biased estimates of N_e_, due to mixture LD [110] caused by combining parents from different cohorts in a single sample [109] but the downward bias tends to be less when the reproductive lifespan approaches the generation length [109]. We also estimated N_e_ using an estimator by parentage assignments (EPA) [111] that has been specifically developed for populations with overlapping generations and is implemented in the software AgeStructure. The EPA is robust to differential fertility among individuals within an age class, disproportional sampling among age classes and errors of age estimation [111]. N_e_ is accurately estimated by EPA using ≥8 microsatellites and when 8–16% of individuals of each age class in the population are sampled [111]. The EPA also provides an estimate of the generation interval for each sex and overall. In AgeStructure, we applied modelled estimates of null allele frequencies and used 95% reliability of parentage assignments, unequal fertility among individuals in both sexes and 1000 bootstrap samples to calculate confidence intervals (CIs). Lastly, we estimated N_e_ using the equation *Θ* = 4N_e_µ, where µ is the mutation rate and a regression corrected homozygosity-based estimator of *Θ* [112]. Microsatellite mutation rates vary greatly among loci [113] but in mammals most of the reported average rates range from 10^−4^ to 10^−3^ mutations per locus per gamete per generation [114,115,116]. Therefore, mutation rates of 10^−4^ or 10^−3^ were used in this analysis.

We tested for recent reduction in N_e_ using Bottleneck 1.2.02 [117]. Significance of heterozygosity excess over all loci, indicative of a recent bottleneck [118], was assessed with sign and Wilcoxon’s signed rank tests [117,119]. Tests were conducted using 10,000 simulation iterations and three different models of microsatellite mutation: the infinite alleles model (IAM), the stepwise mutation model (SMM) and the two-phase model (TPM). For the latter, the variance for mutation size was set to 12 and the proportion of single-step mutations was set to either 95% or 78% as recommended by Piry et al. [117] and Peery et al. [120], respectively. Also, in Bottleneck, we performed the mode-shift test of the shape of the allele frequency distribution, which is expected to be L-shaped under mutation-drift equilibrium [121]. We further tested for a reduction in N_e_ by calculating the mean ratio of the number of alleles to the range in allele size across loci (*M*) [122], which is expected to decrease when a population is reduced in size. The value of *M* and critical values for significance (*M_c_*) were computed using, respectively, the software M_P_Val and Critical_M (available at https://swfsc.noaa.gov/textblock.aspx?Division=FEDandid=3298). *M_c_* values were obtained from 10,000 simulation replicates of microsatellite loci under the TPM in a population at mutation-drift equilibrium. We ran simulations with two values of *Θ* (=4N_e_µ), 0.1 and 10 and assuming either 90% single-step mutations and an average size of multistep mutations (Δ_g_) of 3.5 repeat units (as used by Garza and Williamson [122]) or 78% single-step mutations and Δ_g_ = 3.1 (as suggested by Peery et al. [120]). To compare results and because both the Bottleneck and *M* ratio tests rely on the assumption that the data represents a sample from a single isolated population, analyses were done separately for the APNR and KNP and overall.

We estimated N_e_ and tested for past demographic change using models of a single population with, respectively, constant size (‘OnePop’ model) and a single past variation in N_e_ (‘OnePopVarSize’ model) [123] and coalescent algorithms for ML estimation implemented in Migraine 0.5.4 (available at http://www1.montpellier.inra.fr/CBGP/software/migraine/index.html). The ‘OnePop’ model of a panmictic population at equilibrium estimates a single parameter, *Θ*. The ‘OnePopVarSize’ estimates mutation-scaled current and ancestral N_e_ (*Θ* and *Θ*_anc_) and the scaled age of the demographic change (*D*). The composite parameter N_ratio_ (=*Θ*/*Θ*_anc_) is useful to determine the nature (contraction or expansion) and extent of the past demographic change. The null hypothesis that no size change occurred (i.e., *Θ* = *Θ*_anc_) is rejected at α = 0.05 if and only if 1 lies outside the 95% CI of the N_ratio_ [123]. The analyses were conducted under a generalized stepwise mutation model (GSM) with estimation of the parameter (pGSM) for the geometric distribution of mutation sizes. In a comparison with Msvar [124], one of the most widely used and evaluated programs to infer past demographic change from microsatellite data [125,126,127], Migraine seems more adapted for analyses using microsatellites because of the implementation of a GSM, a more realistic mutation model than the SMM and showed more power to detect past population contraction and lower false expansion detection rates [123]; Msvar estimates may be particularly unreliable when realistic deviations from the assumed SMM occur [125]. To convert estimates of scaled parameters (*Θ* and *D*) into canonical parameters (N_e_ and time), we again used mutation rates of 10^−4^ or 10^−3^, as well as a generation time of 17 years [33]. The analyses were run using 800 points and 20,000 runs per point in each of four (for the ‘OnePop’ model) or eight (for the ‘OnePopVarSize’ model) iterations. For comparison, the analyses were performed both for the APNR alone and for APNR + KNP.

Past changes in N_e_ were also explored using an approximate likelihood method [127] implemented in the R package VarEff 1.2. Its authors stated that among its advantages over Msvar are the facts that the results are less dependent on priors and that different mutation models can be applied [127]. We modelled demographic changes during the last 3000 generations (parameter GBAR) under a SMM or a TPM with 78% single-step mutations and assuming an average mutation rate of 10^−4^ or 10^−3^ mutations per locus per gamete per generation. The fit to the data of models with different mutation processes and rates was assessed based on the mean value of the quadratic deviation of data from the model [127]. The parameter defining the maximum analysed distance in repeat units between alleles (DMAXPLUS = DMAX + 1) was set to 11. We chose this range because 10 was the maximum distance observed with a frequency ≥0.005 and did not lead to the inclusion of distances with zero frequency, the latter of which would violate the assumption of the model that the mean distance frequency is normally distributed [127]. As suggested by Nikolic and Chevalet [127], we used a prior value of N_e_ (parameter NBAR) equal to *Θ_1_*/4µ and a value of three for the variances of the prior log-distributions of both N_e_ (parameter VARP1) and time intervals during which the population is assumed of constant size (parameter VARP2). The prior correlation coefficient between successive population sizes (parameter RHOCORN) was set to zero and the Markov Chain Monte Carlo (MCMC) simulations used 10,000 batches of length 10 sampled every 10 iterations, after a burn-in of 10,000 steps. We tested two to five population size changes (parameter JMAX) and, for each combination of mutation rate and mutation model, we focused on the results of the analysis yielding the most similar estimates of N_e_ from medians and harmonic means of posterior distributions. In order to detect significant changes in population size, we calculated the ratio (RN) [127] between range and mean of N_e_ estimates for a period under consideration; calculations were based on the medians of posterior distributions. An RN value >0.1 is a good indicator of a significant change in population size over the period examined. Again, the analyses were performed both for the APNR alone and for APNR + KNP.

## 3. Results

### 3.1. Microsatellite Data Validation

The mean allelic dropout rate across loci was 0.037, the false allele rate was 0.018 and the overall rate per single locus genotype of the other five types of errors detected by Gimlet was 0.014. Overall, the mean error rate per locus, the most universal metric of genotyping error [128], was estimated at 0.056. The probabilities of identity among the 18 autosomal microsatellite loci were low (unbiased P_ID_ = 2.15 × 10^−15^; P_ID-SIB_ = 1.14 × 10^−6^). The observed distribution of genotypic differences obtained with MM-Dist was bimodal, with the lower mode at 1-MM (one locus mismatch between pairs of samples) and the antimode at 3-MM. The expected probability of two full siblings differing at one, two or three loci was estimated by MM-Dist at 2.4 × 10^−5^, 2.4 × 10^−4^ and 1.5 × 10^−3^, respectively. Thus, being conservative and given our estimate of genotyping error, we considered samples as originating from the same individual if their genotypes differed at ≤2 loci [129]. All samples separated by such genotypic differences were of the same sex. The cumulative P_ID-SIB_ for the 16 loci with the highest P_ID-SIB_ per locus was 10^−5^. With a large number of loci and a per locus genotyping error rate of 5%, the entire lower mode of the bimodal distribution of observed pairwise mismatches is likely due to genotyping error [129]. The probabilities of identity for the 18 loci were recalculated after collapsing matching and nearly matching genotypes (i.e., 0-MM, 1-MM and 2-MM pairs) and results did not change (P_ID_ = 2.41 × 10^−15^; P_ID-SIB_ = 1.12 × 10^−6^). As a result of the genotype comparisons, the 360 samples collected in the APNR were estimated to represent 248 different individuals. With the 46 KNP samples, our dataset included a total of 294 elephants, of which 98 were females (79 adults and 19 juveniles) and 196 were males (151 adults and 45 juveniles) (Supplementary Table S1). The total amount of missing genotype data was 0.94% distributed over seven of the 18 loci, with a maximum of 12 (4.1%) missing genotypes per locus (LaT06 and LaT18). The cumulative P_ID_ and P_ID-SIB_ for the 11 loci without missing data were 4.6 × 10^−9^ and 3.6 × 10^−4^, respectively. Of the 294 animals analysed, only 34 had missing data and this only for one locus in 25 of them; the maximum number of loci with missing data per individual was four (in two individuals). The H-ind analysis in GenAlEx gave a mean heterozygosity of 0.62 ± 0.12. The lowest observed H-ind was 0.29 and only nine and 36 individuals had H-ind lower than 0.4 and 0.5, respectively.

Two different sets of samples were used to assess the quality of the microsatellite data. After a sequential Holm-Bonferroni correction with a nominal α = 0.05, significant deviations from Hardy-Weinberg equilibrium (HWE) and linkage equilibrium (LE) were observed, respectively, for three loci (FH67, LaT06 and LaT25) and between two pairs of loci (FH39 and LaT08 and FH40 and LaT18) in the analyses including all APNR individuals but no deviations were found when using a subset of unrelated individuals (Supplementary Table S2). The lack of consistent departures from HWE and LE suggest that the observed deviations are intrinsic to the sample set analysed and that the data are not significantly affected by locus-specific problems.

Micro-Checker indicated that null alleles might be present at loci FH71, LaT06, LaT13 and LaT25, with null allele frequencies of 0-0.06, 0.08-0.10, 0.02-0.04 and 0.08-0.12, respectively, as estimated by the different null allele frequency estimators used. Both ML-NullFreq and PIM in Inest indicated null alleles at a further locus, FH67, with frequency 0.04 (0.03 by the Van Oosterhout algorithm, Cervus and IIM) (Supplementary Table S3). In the IIM, the DIC selected a model including null alleles and genotyping failure but not inbreeding. Estimates of genotyping failure rates for each locus from both ML-NullFreq and Inest ranged from 0 to 0.02. Importantly, null allele frequencies per locus <0.1 are unlikely to have a significant impact on the results of population genetic analyses [130,131,132,133].

### 3.2. Genetic Diversity and Structure

All of the 18 microsatellite loci were polymorphic, with 3 to 14 alleles per locus. The observation of heterozygotes in both males and females confirmed that all loci were autosomal. Mean observed and expected heterozygosities across loci were, respectively, 0.63 and 0.64 for the APNR and 0.60 and 0.63 for KNP. The mean allelic richness across loci was also similar between the two areas (6.25 for APNR and 6.04 for KNP), with private allelic richness being slightly higher in the APNR (0.89) than in KNP (0.68). The mean values of F_IS_ over all loci were statistically significant and positive but low for both the APNR and KNP, being slightly higher in the latter (Table 1). The observed F_IS_ values may not be related to inbreeding, as this should equally affect the F_IS_ values for all loci in each sample but instead be due to null alleles, allelic dropout and fine-scale genetic structure [57,134,135].

**Figure 2:**
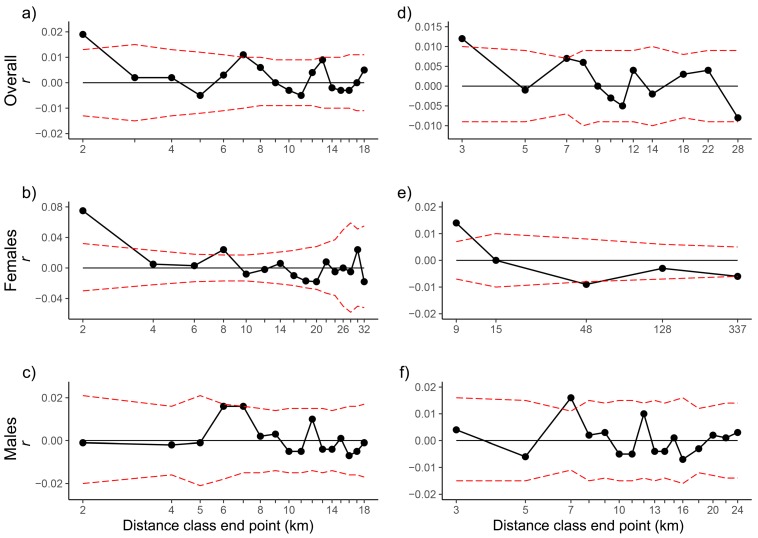
Spatial autocorrelation correlograms for all APNR + KNP adult samples - (**a**) and (**d**) - and separately for each sex (females: (**b**) and (**e**); males: (**c)** and (**f**). *r* is the autocorrelation coefficient, a measure of the genetic similarity between pairs of individuals falling within a given distance class. The left plots show results using the same upper bound of 2 km for the first distance class and the right plots show results using a first distance class such that, for all adults and for adult females only (top two plots), it reveals the approximate geographic distance up to which positive spatial genetic structure was detectable. All distance classes in the correlograms contained more than 100 pairwise comparisons. The red dashed lines represent the 95% confidence envelope for the hypothesis of no spatial genetic structure as determined by permutation.

The SAC analyses including both sexes indicate positive and statistically significant fine-scale genetic structure up to 2–3 km (Table 2; Figure 2). The correlograms with a distance class size of 2 km had the first x-intercept for *r* at 4.3 km. In females, a significantly greater genetic similarity between individuals than expected by chance was detected in analyses using a first distance class of 8–9 km; in the test at the APNR level with a first distance class of 9 km, the lower bootstrap 95% confidence limit was 0. The correlograms with a distance class size of 8 km for the APNR and APNR + KNP had the first x-intercept for *r* at 12.8 and 14 km, respectively. In males, in contrast, the hypothesis of a random distribution of genotypes was not rejected even within 1 km distances. The heterogeneity test of Smouse et al. [70], using a significance level of 0.01 as recommended by Banks and Peakall [71], confirmed the overall significance of the correlograms for all analyses performed except those for the APNR males (Table 2). In the direct comparisons between the sexes, the ω test did not detect heterogeneity between their correlograms in the various analyses performed but the squared paired-sample *t*-test statistic (*t*^2^), with a significance level of 0.01, indicated significant differences in spatial genetic structure using a first distance class of 2 km. In the analyses using only the APNR adult samples from the second (dry) season, a significant fine-scale genetic structure was detected in females (up to 2 km) but not in males and combined sexes; in the direct comparison between the sexes, significant differences were detected with a first distance class of 2 km by the *t*^2^ test.

The Mantel tests of IBD were either not significant (*p* values > 0.05) or yielded very low correlation coefficients (*r_XY_* ≤ 0.02), depending on the measure of genetic similarity/dissimilarity used (Supplementary Table S4; Supplementary Figure S1). Similar patterns were observed in the analyses for males only, whereas females showed a stronger relationship between kinship and geographic distance (*r_XY_* ≤ 0.09). While the tests using Rousset’s distance were all non-significant, those using Nason’s kinship coefficient tended to be significant. Non-significant results were obtained in the analyses using only the APNR adult samples from the second (dry) season.

**Figure 3:**
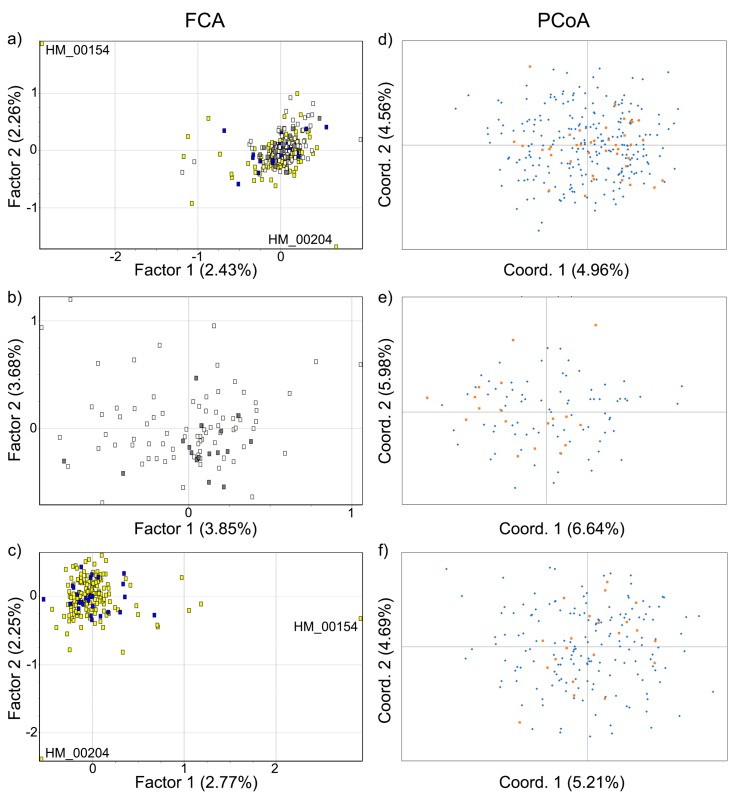
Factorial correspondence analysis (FCA) and principal co-ordinates analysis (PCoA) two-dimensional plots of microsatellite variation of elephants in the APNR and in KNP. (**a**) and (**d**): all individuals; (**b**) and (**e**): all females; and (**c**) and (**f**): all males. In the FCA plots, APNR males are in yellow, KNP males are in blue, APNR females are in white and KNP females are in grey. The two outlying males are labelled. In the PCoA plots, blue represents individuals from the APNR and orange represents individuals from KNP. In the FCA and PCoA plots, respectively, the percentage of the total inertia accounted for by each of the first two factors and the percentage of variation explained by each of the first two coordinates are given in parentheses.

**Figure 4:**
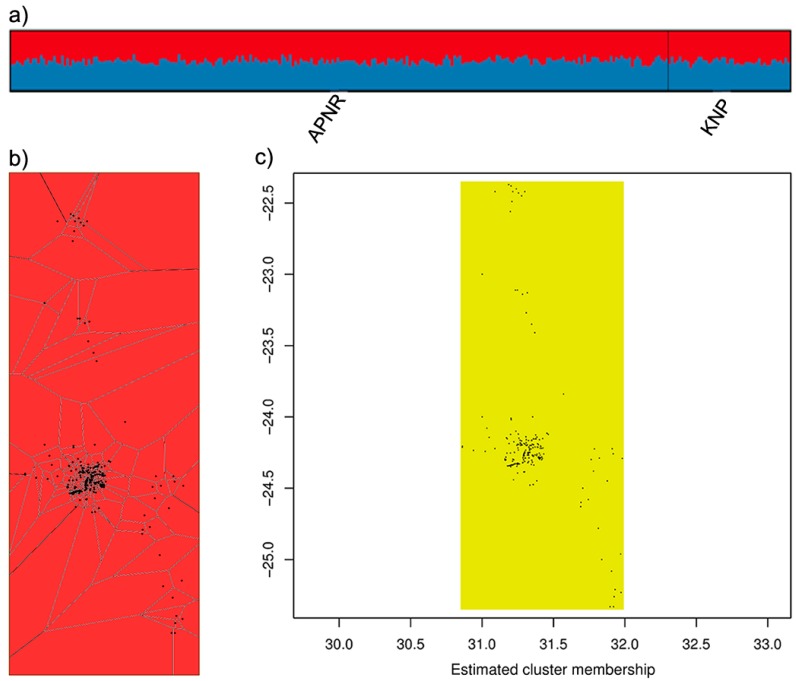
Bayesian clustering results for all individuals in the APNR and KNP. (**a**) Bar plot from Structure when *K* = 2. Each individual is depicted by a column that is partitioned into K segments, which length is proportional to the membership coefficient of the individual for each cluster. A vertical black line separates individuals from the two areas, which are labelled below the figure. (**b**) Voronoi tessellation map for the most supported *K* = 1 in the no-admixture model of Tess. (**c**) Map of estimated cluster membership for the most supported *K* = 1 under the uncorrelated allele frequency model in Geneland. The dots in (**b**) and (**c**) represent the sampling location of individuals.

The ordination analyses indicated a lack of genetic differentiation between APNR and KNP (Figure 3). The apparent genetic diversity of the APNR elephants is noteworthy, given the size of the area in relation to the KNP. In general, the first two axes explained only a small proportion of the total variation but the FCA of allelic composition identified two outlying individuals (two uncollared males in the APNR with identification numbers HM_00154 and HM_00204 (see Supplementary Table S1; Figure 3a,c). Most of the Bayesian clustering methods (the admixture model with correlated allele frequencies in Structure, the uncorrelated allele frequency model in Geneland and the no-admixture and CAR admixture models in Tess) also grouped all individuals from the APNR and KNP into a single genetic cluster, in analyses overall and separately for each sex (Figure 4; Supplementary Figure S2). On the other hand, both the correlated allele frequency model in Geneland, regardless of the *β* prior used and the BYM admixture model in Tess were consistently unable to find a stable *K* solution for any of the three sets of individuals. Moreover, in each of these two models, when independent runs converged on the same value of *K*, they suggested different groupings of individuals. The analyses in Tess and Geneland using only the APNR samples from the second (dry) season also did not find any consistent evidence of genetic structure.

The exact G-test indicated significant (α = 0.05) genetic differentiation between APNR and KNP in analyses including all individuals (*p* value = 0.015), only adults (*p* value = 0.038) or only adult females (*p* value = 0.016) but not when only adult males were considered (*p* value = 0.288). Likewise, estimates of Fst and related measures between APNR and KNP were significant, based on a permutation test, in comparisons involving all individuals (*p* value = 0.008), only adults (*p* value = 0.014) or only adult females (*p* value = 0.001) but not in those for adult males only (*p* value = 0.669). However, only for the adult females the 95% bootstrap CIs did not straddle zero and mean values were not very small: Fst = 0.014, G’’st = 0.039 and Dest = 0.025 (Table 3). Accordingly, the permutation test of Goudet et al. [95] supported the hypothesis that Fst is significantly higher in adult females than in adult males (*p* value = 0.007).

### 3.3. Effective Population Size and Demographic History

The LD and EPA methods gave point estimates of contemporary N_e_ for the APNR and APNR + KNP ranging approximately from 200–400 and from 500–700, respectively (Table 4). The respective N_e_ estimates from *Θ_F_* [112], assuming an average microsatellite mutation rate of 10^−3^ mutations per locus per gamete per generation, were comparable to each other (*Θ*_APNR_ = 2.474 and *Θ*_APNR+KNP_ = 2.459, both of which translate into a N_e_ of ~ 600) and within the ranges of the estimates from the other two methods. Alternatively, accepting a mutation rate ten times lower translate into N_e_ estimates one order of magnitude higher (Table 4). The estimator *Θ_F_* tends to overestimate the true value of *Θ* when mutations follow the GSM instead of the SMM but the bias is small when *Θ* is small (<5) [112]. A trial analysis of the KNP data with the LD method gave an infinite estimate of N_e_ (lower 95% confidence limit of 239.1 but infinite upper bound), indicating insufficient sample size to capture the signal of genetic drift. Therefore, we did not estimate N_e_ for the KNP data alone. We also attempted to estimate the effective number of breeders (N_b_), based on the APNR samples organized into 10-year pooled ‘cohorts’ to ensure exclusion of parent-offspring pairs and reasonable sample sizes (30–70) [136,137] but the LD N_e_ estimates for the four constructed pooled ‘cohorts’ (–10, 10-20, 2–30, 3–40 years) all had 95% CIs with infinite upper bounds. This may be due to a large N_e_, so that the sample sizes available for each ‘cohort’ were not sufficient to obtain reliable estimates [100]. The EPA estimated for the APNR and APNR+KNP a paternal generation interval (GI) of 21.93 years (95% CI: 14.41–32.30) and 14.52 years (95% CI: 13.35–27.51), a maternal GI of 13.83 years (95% CI: 9.67–20.54) and 13.66 years (95% CI: 10.17–20.82) and a mean GI of 17.88 years (95% CI: 13.58–24.50) and 14.09 years (95% CI: 12.92–22.01), respectively. The estimated average GI from the APNR data agrees with that obtained in a demographic study of a savannah elephant population that has been relatively unaffected by poaching during the last four decades (17.38 years, Amboseli National Park) [33]. Importantly [111,138], in both the LD and EPA analyses, random mating was assumed and this was supported by the absence of or weak deviations from panmixia suggested by the results of the IBD tests and the ordination and Bayesian clustering analyses. In species living in social groups and with high rates of dispersal in at least one sex, a deme may behave similar to random mating so that the social structure has little effect on N_e_ [139,140].

**Figure 5:**
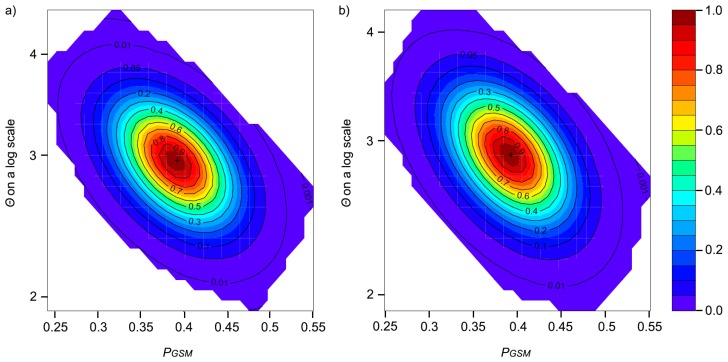
Two-dimensional profile likelihood regions of the parameter for the geometric distribution of mutation sizes (pGSM) and *Θ* from Migraine’s ‘OnePop’ model for: (**a**) APNR and (**b**) APNR+KNP. pGSM and *Θ* in log scale are in the x and y axes, respectively.

The Migraine analyses using the ‘OnePop’ model yielded highly similar point estimates and 95% CIs of *Θ* and pGSM for the APNR and APNR+KNP (Figure 5). The approximate values of *Θ* and pGSM were, respectively, 2.9 (95% CI: ~ 2.3–3.6) and 0.39 (95% CI: ~ 0.30–0.47). The former values translate into N_e_ estimates of around 725 (95% CI: ~ 575–900) or 7,250 (95% CI: ~ 5750–9000) assuming an average mutation rate of 10^−3^ or 10^−4^, respectively (Table 4). A trial analysis using only the smaller KNP data set, which may be insufficient for reliable inference in Migraine [123], also gave estimates of *Θ* and pGSM highly similar to those above: 2.8 (95% CI: 2.1–3.6) and 0.36 (95% CI: 0.25–0.46). The pGSM estimates support the overall loci inadequacy of the SMM for the data. In the ‘OnePopVarSize’ model, the estimates of *Θ* and pGSM for the APNR (2.9, 95% CI: 2.3–3.6; 0.36, 95% CI: 0.26–0.45), KNP (2.6, 95% CI: 2.0–3.5; 0.31, 95% CI: 0.19–0.42) and APNR+KNP (2.8, 95% CI: 2.3–3.5; 0.35, 95% CI: 0.26–0.45) were also similar between data sets and to those from the ‘OnePop’ model. The ‘OnePopVarSize’ model analyses of the three data sets all hinted at strong past population contraction (N_ratio_ point estimates of 5 × 10^−4^ – 5 × 10^−3^, that is, << 1) but without statistical significance (95% CIs ranging in order of magnitude from −5/−4 to 3/5). The point estimates of *Θ*_anc_ were very large, ranging from 572 for the APNR to 5290 for APNR+KNP, which assuming mutation rates of 10^−3^ or 10^−4^ translate into unrealistically large N_e_ estimates of 1.4−13.23 × 10^5^ or 10^6^, respectively; the 95% CIs of *Θ*_anc_ had upper bounds that could not be estimated (Supplementary Figure S3), a likely outcome when no obvious past demographic change could be detected [141]. The estimates of the timing of population contraction were also unrealistically ancient: 5–7 × 10^5^ or 10^6^ years ago assuming mutation rates of 10^−3^ or 10^−4^, respectively; again, the 95% CIs of *D* were very wide, ranging in order of magnitude from −2/0 to 4/5 (Supplementary Table S5). The non-significant signal of a strong ancient contraction inferred by the ‘OnePopVarSize’ model may reflect the fact that the elephant population in the study area was part of a historical structured metapopulation [142]. Moreover, the overall Migraine results indicate that APNR and KNP belong to the same population and share a common history.

**Figure 6:**
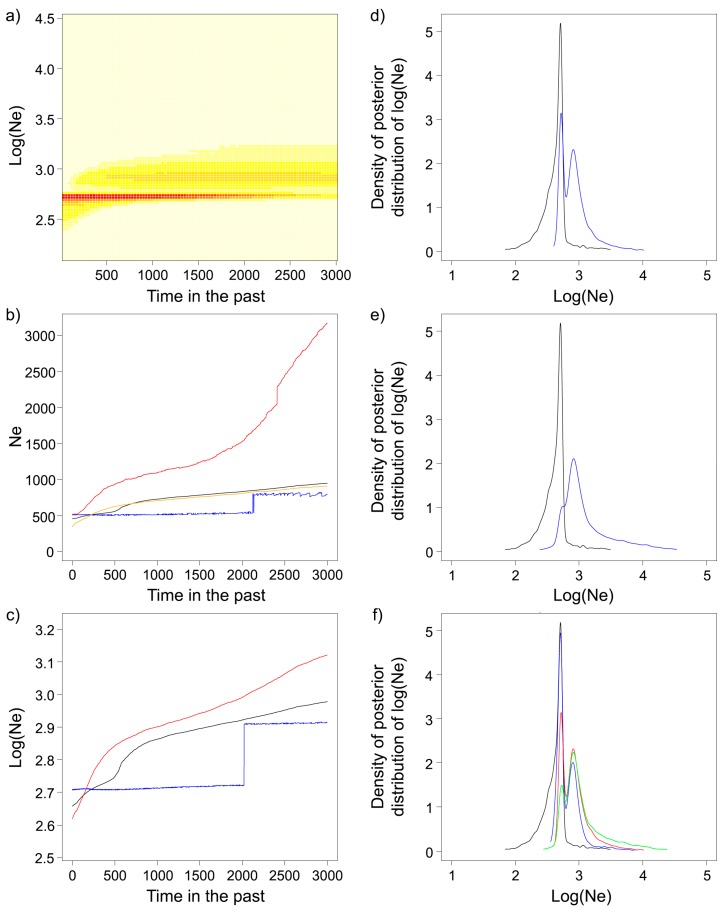
VarEff inference of demographic history over the last 3000 generations based on the APNR data set. (**a**) Joint posterior distribution for logN_e_ (*y*-axis) over time in generations (*x*-axis); (**b**) and (**c**) mean (red), median (black), mode (blue) and harmonic mean (orange) of N_e_ estimates in linear and logarithmic scales, respectively; (**d**), (**e**) and (**f**) densities of the posterior distributions of logN_e_ for the present and at different times in the past. (**d**) logN_e_ estimates for the present (black) and 1500 generations ago (blue), (**e**) logN_e_ estimates for the present (black) and 3000 generations ago (blue) and (**f**) logN_e_ estimates for the present (black) and 500 (blue), 1500 (red) and 2500 (green) generations ago.

In the VarEff analyses for both the APNR alone and for APNR+KNP, the model best-fitting the data (i.e., the one with the smallest mean quadratic deviation of observations from the model) assumed TPM mutations at a rate 10^−3^ and two past changes in N_e_. This model also yielded the most similar estimates of N_e_ from medians and harmonic means of posterior distributions. A test analysis using only the KNP dataset, again bearing in mind the possible inadequacy of sample size, had the same best-fit model. The two main analyses (APNR and APNR+KNP) inferred virtually identical demographic histories and indicated a gradual decrease in N_e_ (RN = 0.66–0.67) over the time period modelled (3000 generations or 51,000 years assuming a generation time of 17 years), translating into about a halving of the median estimates of N_e_ (e.g., for the APNR, median N_e (T = 0)_ = 455 and median N_e (T = 3000)_ = 951) (Supplementary Tables S6 and S7). When dividing the whole period into 12 intervals of 250 generations, to further understand the demographic pattern, the RN ratio results only suggested significant changes in N_e_ during two time spans: 500 to 750 generations ago (RN = 0.19–0.21) and in the last 250 generations (RN = 0.12–0.13). Further analyses of the latter period showed that a significant decrease in N_e_ could still be observed when only the last 200 generations were considered (RN = 0.10–0.11) but not in the last 150 generations (RN = 0.08–0.09). The probability of the time to the most recent common ancestor (TMRCA) between two alleles being less than 500 and 250 generations was estimated at 0.4 and 0.25, respectively. Comparison of plots of the density of the posterior distribution of log(N_e_) at different times showed that only those for generations at the extremes of the 0–3000 interval were separated, with partial overlap (Figure 6 and Supplementary Figure S4). However, when considering the corresponding 90% central ranges (CR) of the N_e_ estimates in natural scale, their overlap is significant even for the comparison between the limits of the 0–3000 interval (e.g., for the APNR, N_e (T = 0)_ 90% CR = [161–971] and N_e (T = 3000)_ 90% CR = [497–11,697]) (Supplementary Tables S6 and S7).

The sign and Wilcoxon’s signed rank tests in Bottleneck under the SMM or TPM, except those using the latter model with 78% single-step mutations (which were not significant at α = 0.05), revealed significant heterozygosity deficiency over all loci in all the three data sets analysed (APNR and KNP separately and pooled together). In contrast, assuming the IAM, most of the tests indicated significant heterozygosity excess over all loci (Supplementary Table S8). The type I error rates of the heterozygosity excess tests are higher under the IAM than the SMM [118,119] and the latter model is considered more suitable for microsatellites [143], with the TPM being probably the most appropriate model overall [117,144]. Therefore, when a significant heterozygosity excess is only obtained under the IAM, it seems reasonable and conservative to conclude that there is no evidence for a recent decline in N_e_. The mode-shift test showed a normal L-shaped distribution of allele frequencies, as expected under mutation-drift equilibrium, in all the three data sets. In the *M*-ratio tests, the mean *M* values observed for the KNP (0.853), APNR (0.905) and the two combined (0.904) were higher than the respective critical values *M_c_* for the four different combinations of *Θ* and mutational processes tested, except in the case of the KNP assuming *Θ* = 0.1, 90% single-step mutations and Δ_g_ = 3.5 (*M_c_* = 0.871) (Supplementary Table S9). This last result was considered spurious because the sample size for the KNP was modest and the *M*-ratio test was significant for a single set of assumed parameters.

## 4. Discussion

In this study, we conducted a conservation genetic assessment of savannah elephants in the Greater Kruger Biosphere, with a focus on those in previously unstudied nature reserves (APNR) adjacent to KNP. Our aims were: i) to characterize the genetic status of the APNR elephant population; ii) to examine genetic structure across the Greater Kruger; iii) to provide estimates of local effective population size; and iv) to investigate demographic history and in particular to test for genetic bottlenecks, which might be expected given the known history of South African elephants.

### 4.1. Genetic Diversity and Structure

The sample sets from the APNR and from across the KNP showed similar and moderate mean observed microsatellite heterozygosity (0.625 and 0.595, respectively). These values are also comparable to those obtained in a previous study [30] for the KNP (0.626), based on a total of 33 samples in two balanced clusters in the north and south of the park and in other areas in Southern Africa [22,30,145]. The average levels of heterozygosity in the APNR or the KNP, however, were in general lower than those of eastern African populations [13,146,147,148]. This pattern may be due to the different marker sets used between studies, since another study that analysed both KNP and eastern African population samples (albeit of small size) using the same microsatellites found similar levels of average observed heterozygosity and number of alleles per locus [22]. Nevertheless, for some of the loci used here and in studies of East African elephants [13,37,146,149], our estimates of heterozygosity and allelic richness in the APNR or the KNP were lower than those reported in eastern African populations (FH67, FH103, LaT06, LaT18, LaT25). Inter-population comparisons of allelic richness at the same microsatellite loci can be powerful, far more so than analogous heterozygosity comparisons, for identifying populations that have undergone a recent bottleneck [150]. Whitehouse and Harley [28] also found lower heterozygosity and allelic diversity at another four loci in the KNP than in three Ugandan elephant populations [151]. They interpreted these results as reflecting the different histories of South and East African elephant populations: while the former suffered severely from hunting and poaching during the eighteenth and nineteenth centuries [25], the latter have been decimated by widespread poaching in more recent times, during the last 50 years [13,18,146,148,151]. Therefore, they suggested that although the Kruger elephant population recovered rapidly from its demographic bottleneck in the early 1900s, it shows signs of the genetic diversity loss caused by the earlier massive and long-term decimation of South African elephants, whereas, given the long life span and generation time of elephants, a significant decrease in genetic variation may not yet be observable in the very recently reduced East African elephant populations. However, no evidence of a recent genetic bottleneck in Kruger elephants has been found by Bottleneck and *M* ratio analyses in this and other studies [28,30]. The *M* ratio method in particular might have been expected to be able to detect a possible recent N_e_ reduction in the Kruger elephants, as it can be powerful for detecting bottlenecks when they were severe and lasted several generations, the population had made a demographic recovery and the pre-bottleneck population size was large [120,152]. The fact that the Bottleneck and *M* ratio tests clearly did not support the hypothesis of a genetic bottleneck may be due to the rapid post-bottleneck growth of the Kruger elephant population [25,28,30] that likely resulted in a less severe overall reduction of N_e_ [149,153], a situation in which for instance the *M* ratio method has less power [120,152]; in turn, the heterozygosity excess test assumes a permanent bottleneck in population size [119] but see Hoban et al. [154]. Concomitantly, immigration of elephants into the Kruger population from Mozambique [25,28] and possibly from other nearby areas in Southern Africa [30], likely brought new alleles, thus erasing signatures that could be detected by the Bottleneck and *M* ratio tests [155]. Also, given the long elephant generation time, the demographic contraction may not have occurred long enough ago to be detected by the Bottleneck and *M* ratio tests [134]. Simulation studies have shown that these tests may have low to moderate power to detect even large reductions in N_e_ when they occurred ≤ 10 generations ago [118,120,126]. Moreover, a long generation time provides a buffer against the rate of loss of genetic variation during short-term bottlenecks [156]. Lastly, the social and mating system of savannah elephants may also have helped to maintain genetic diversity, as different alleles could have survived in different social groups [57,139]. A combination of rapid post-bottleneck population growth, immigration, long generation time and social structure and mating system, may then explain why the known recent demographic bottleneck is not reflected in a significant signature of genetic bottleneck. A possible test of whether the demographic collapse during the 19th–20th century transition caused a genetic bottleneck could be the comparison of levels of genetic diversity and N_e_ between samples from the Kruger area from the 1920s–1930s (after the demographic contraction but before the population rebound) and the mid-19th century [25], if such specimens are available.

The exact G-test indicated significant genetic differentiation between APNR and KNP but the results of genetic structure analyses using several different methods of ordination and Bayesian clustering strongly suggest that elephants throughout the GKNP constitute a single population. Concordantly, although estimates of F_st_ and related measures between APNR and KNP were significant based on permutation, their values were very small (0.004–0.011) and the 95% bootstrap CIs included zero. Moreover, Mantel tests of IBD, using different inter-individual genetic distances, were either not significant or yielded very low correlation coefficients (*r_XY_* ≤ 0.02). Overall, these different results indicate a lack of genetic structure across the GKNP. It has been shown that exact tests using microsatellite data can be very powerful in detecting even very small departures from panmixia [89,157] but such powerful tests can also yield statistically significant results that do not reflect biologically meaningful genetic differentiation [157]. Our genetic structure results agree with the study of de Flamingh et al. [30], which concluded a lack of microsatellite differentiation between two sample sets from north and south KNP.

The SAC analyses including both sexes indicated positive and statistically significant fine-scale genetic structure up to 2–3 km. Fine-scale structure can exist even when dispersal is restricted in only one sex [158,159], while gene flow mediated by the dispersing sex may explain the lack of genetic structure at larger spatial scales [140,159]. The detected fine-scale structure is the result of female matrilocality and the presence of family groups [57]. With sex-biased dispersal, the philopatric sex is expected to exhibit stronger and more extensive fine-scale structure than the dispersing sex, which may show an apparently complete absence of fine-scale structure [71]. Accordingly, given the male-biased dispersal known for elephants [57,151], the SAC analyses for females indicated significantly positive genetic correlations among individuals up to 8–9 km apart, whereas for males the hypothesis of a random distribution of genotypes was not rejected even within 1 km distances. Also, fittingly, the genetic differentiation tests (G-test and F_st_ and related statistics) between APNR and KNP were significant for females but not for males. The weaker fine-scale genetic structure observed in the SAC analyses using only the APNR adult samples from the second (dry) season may potentially be due to the contraction of elephant home ranges in the dry season [32,160]. Therefore, the inferred patterns in the SAC analyses with samples from the entire sampling period, corresponding to approximately two wet seasons and one dry season, may mainly reflect the fine-scale genetic structure in the wet season. The extent of spatial genetic structure up to 8–9 km in females may be an underestimate given the use of the bootstrap confidence interval test, which is more conservative than the permutation test [67]. In any event, to our knowledge, this is the first estimate of the scale of spatial genetic structure in female savannah elephants. This information is valuable for behavioural and ecological research and conservation management [17,57]. For example, for comparisons of patterns among elephant species and populations and between protected and unprotected areas [161]. Fine-scale genetic structure in female elephants is important for population health but is impacted by poaching and other human activities [13,17,57] and therefore may differ between populations under different levels of anthropogenic pressure.

### 4.2. Demographic History and Effective Population Size

Coalescent-based likelihood estimates in Migraine and VarEff also did not support the hypothesis of a significant N_e_ reduction in the recent past. The Migraine analysis using the ‘OnePopVarSize’ model yielded a non-significant signal of a strong ancient decrease in N_e_. In turn, VarEff inferred a monotonic decline in N_e_ during the last ~50,000 years, with two steeper drops 12750–8500 years ago and 4250–3400 years ago, resulting overall in noticeably lower point estimates of contemporary N_e_; however, the 90% CR of the N_e_ estimates overlapped substantially through time, even between today and 50000 years ago. Thus, neither Migraine nor VarEff analyses could find unambiguous and statistically significant evidence for past demographic changes. The non-significant signal of a strong ancient population contraction inferred by the ‘OnePopVarSize’ model of Migraine may reflect the fact that the Kruger elephant population was part of a historical metapopulation [142]. It is known that population structure of the island model type [96] can generate lineage genealogies that resemble those resulting from population bottlenecks [162] and hence may confound analyses of demographic history [163]. Given the massive human impacts over the last centuries on the Southern African elephant populations, these are unlikely to be in migration-drift equilibrium and recent and current levels of interpopulation gene flow are unknown. Island model simulations suggest that when individuals are sampled from a single deme, Migraine may have moderate false contraction detection rates if the N_e_ of demes is neither small nor large (a few hundred) and migration rates between demes are low (≤1%), so that the genealogy of a sample taken from a single deme is closer to that expected in a Wright-Fisher population [123]. On the other hand, in similar island model simulations but in which populations have undergone a past reduction in N_e_, sampling a single deme resulted in low contraction detection rates; these increased when levels of gene flow were higher [123]. VarEff is also sensitive to gene flow and the demographic trend inferred by VarEff might reflect recurrent immigration at low to moderate rates (see Figure 7 in Nikolic and Chevalet [127]).

The effective size of a population is a key parameter in evolutionary and conservation biology as it determines the equilibrium level of neutral or weakly selected genetic variability, the rate of genetic drift and inbreeding, the relative importance of migration and the effectiveness of selection [164,165]. In conservation contexts, genetic estimates of short-term N_e_ are important to understand current rates of loss of genetic variation and increase of inbreeding, vulnerability to genetic stochasticity and therefore are essential for assessing the status and viability of a population under current management and habitat conditions and its capacity to respond and adapt to environmental challenges and changes [166,167,168]. Estimates of long-term N_e_ offer a retrospective view of the rate of genetic drift over past generations and, when compared with those of current N_e_, may be useful in determining whether a population has undergone a recent demographic change [134,156,166]. Although estimates of long-term N_e_ are often equated with N_e_ over many generations, when they concern recently fragmented or bottlenecked populations and especially if based on microsatellites, they are more likely to reflect recent effective sizes [166]. The LD and EPA estimators of short-term N_e_ gave higher point estimates of N_e_ for APNR + KNP (500–700) than for the APNR alone (200–400) but the respective 95% CIs overlapped substantially. To our knowledge, these are the first available estimates of N_e_ for Kruger’s elephants. Given our relatively limited sample size for the KNP, a better sampling of this area should be obtained for N_e_ estimation. The LD estimates of N_e_ may be underestimated due to several factors, all of which may have had an impact. Firstly, the underlying model of the LD method assumes discrete generations, with the estimated N_e_ referring to the parental generation of the sample [169]. When samples are taken from age-structured species, which can introduce non-random mating effects, the estimate from the LD method can be an estimate of the effective number of breeders (N_b_) that produced the cohort(s) from which the sample was taken or of a quantity intermediate between N_b_ and N_e_ [100,109,169]. It has been shown that using only adults is preferable to using all individuals in the population for estimating N_e_ from LD when generations overlap [108,109] and therefore we used only adults but this strategy may still lead to an underestimation of N_e_, which in several species studied was about 20–30% when the ratio of adult life span (=maximum age – age at maturity + 1) to generation length is in the range 2–3 [109]; in savannah elephants, assuming a maximum age of 65 years, a mean age at first reproduction of 14 years and a generation time of 17 years [33], the ratio in question is 3. However, it is important to note that the comparisons in Waples et al. [109] are between LD estimates and analytical expectations of N_e_ based on a demographic model assuming a population of constant size [170], a scenario that does not apply to Kruger elephants. Secondly, both the LD and EPA methods assume a population of constant size. The consequences of violating this assumption have not been evaluated for the EPA but a recent change in population size can cause LD N_e_ estimates to be affected by N_e_ in generations prior to the one sampled [97]. For unlinked loci, a recent population decline is unlikely to seriously affect LD estimates of N_e_ because the signal from current (reduced) N_e_ is strong relative to the signal from larger N_e_ in previous generations but if the population has recently increased, as is the case of the Kruger elephants, LD estimates of N_e_ can be biased downwards for a few generations, with duration and magnitude of bias proportional to the severity of the previous population reduction and relative increase in N_e_ [97,169]. Finally, thirdly, the LD method can be very sensitive to positive values of F_IS_, potentially substantially underestimating N_e_ even for low values of F_IS_ (e.g., 0.02–0.05; [138]), that is similar to those estimated here for the APNR and KNP samples (Table 1). The EPA assumes random mating but its robustness to deviations from this assumption has not yet been tested. Moreover, immigration of genetically differentiated individuals from other populations leads to mixture disequilibrium [110,171] that could downwardly bias LD estimates of local N_e_ [100]. It has been shown that under equilibrium migration models this effect is small but nonequilibrium pulse migration of strongly divergent individuals can create strong mixture LD and depress estimates of local N_e_ [103]. Nevertheless, this second scenario does not appear to apply in our data set because neither the Bayesian clustering nor the ordination analyses, except the FCA that identified two outlying males in the APNR, indicated the presence of recent immigrants that could have strongly influenced estimates of N_e_. Overall, despite the aforementioned potential reasons for a possible underestimation of the LD estimates of N_e_, the fact that these are similar to the EPA estimates and considering that the LD and EPA methods are based on very different approaches to estimating N_e_, can be seen as increasing the confidence in the estimates [100,172].

Comparable N_e_ estimates to those obtained by the LD and EPA methods were inferred from analyses in Migraine (~700), VarEff (~400) and *Θ_F_* (~600), in all three cases assuming an average mutation rate of 10^−3^. The analyses in VarEff suggest that this rate fits the data better than a rate of 10^−4^ and it agrees well with reported overall average mutation rates for mammalian tetranucleotides [116,173]. However, a value of 10^−3^ can be considered as in the upper bound estimate of the average microsatellite mutation rate in mammals and the typical average rate, in particular for dinucleotide repeats, is probably somewhat lower [115,173]; assuming, for example, a mid-range value of 5 × 10^−4^, a figure often used in the literature (e.g., Reference [174]), would translate into a doubling of the N_e_ estimates from Migraine (~1400), VarEff (~800) and *Θ_F_* (~1200). On the other hand, since the microsatellites used in this study were selected for their polymorphism, their mutation rates may actually on average be higher than the mean mutation rates observed in microsatellite panels unfiltered for polymorphism [166]. In any case, it is interesting that the N_e_ estimates from these different methods are comparable, given their differences in model assumptions and estimation algorithms. For instance, mutation-drift equilibrium is assumed by *Θ_F_* but not by VarEff. However, all three methods assume an isolated population. It is well known that even low levels of immigration may strongly influence genetic diversity in local populations and this can lead to biased estimates of local N_e_ by *Θ*-based N_e_ estimators [107,134,166]. The risk of a possible substantial overestimation of local N_e_ that even a small amount of immigration can cause has been illustrated for the island model [175]. However, the island model assumption of constant and identical immigration rates from different source demes may be particularly unrealistic for the Kruger elephants. Instead, the bulk of immigrant gene flow into the Kruger elephant population may have come and continue to come, from the adjacent LNP. Recurrent immigration of elephants into the Kruger from neighbouring Mozambique may have been substantial during 1960–1970, due to heavy hunting pressure and drought in that country [25] and then possibly again since the early 2000s when fences between KNP and LNP began to be removed, ending nearly three decades of separation [31]. This gene flow into the Kruger may imply that our long-term N_e_ estimates could be biased upwards. Nevertheless, the magnitude of this bias may be limited. The genetic structure analyses did not consistently indicate the presence of genetically divergent immigrants that could have strongly affected the N_e_ estimates. Moreover, the effect of immigration on estimates of local N_e_ may be modest if allele frequencies are similar between the focal and source demes, so that immigrants have less effect on allele frequency changes [168,176]. A future study of the elephant populations in the LNP and other areas of Mozambique, using the same microsatellites as here, would allow, among other objectives, to compare the populations in the LNP and Kruger and test the hypothesis of their genetic similarity. A regional sampling, also including other potential sources of immigrants to Kruger in Zimbabwe and Botswana, would additionally allow estimation of N_e_ using methods that account for migration (e.g., References [177,178]).

The LD and EPA point estimates of contemporary N_e_ for the GKNP were 500–700. These estimates are close to values of N_e_ that have been proposed as absolute minimum for the long-term maintenance of quantitative genetic variation and evolutionary potential of a population (N_e_ = 500–1000) [179,180,181,182,183]. Clearly, the current census size of a population, which in the case of the GKNP elephants is estimated at about 21,000 individuals [26], can give a misleadingly optimistic impression of population viability [184]. Considering the ratio of N_e_ to population size is important because if this ratio is very small (<0.1), then genetic drift can be strong although the population is large [185]. The LD and EPA estimates provide information about N_e_ in the population of breeders that produced the sample; therefore, to estimate N_e_/N, we used as denominator the mean of the estimated number of adult elephants (N_a_) [165,183,185,186] in the GKNP, assuming a stable age structure with 38% of mature individuals [26], over the period 1905–1990 [25]. We also calculated N_e_/N using the mean census size (N_c_) over the same period in the denominator. The ratio N_e_/N_c_ is directly proportional to the total reproductive value of a population [187] and gives an idea of the relative risks that demographic and genetic factors might pose to its persistence in the short term [188]. Based on the range of the LD and EPA point estimates of N_e_, the values obtained for N_e_/N_a_ and N_e_/N_c_ were 0.27–0.37 and 0.10–0.14, respectively. These ratios, however, may be overestimated because, when temporal trends in N are evident (in the case of GKNP elephants, a strong population growth during the 20th century, particularly from 1960 onwards), it may be more appropriate to use the more recent values of N to estimate the denominator of N_e_/N [186,189]. Using the mean N_a_ and N_c_ for the period 1960–1990, the N_e_/N_a_ and N_e_/N_c_ ratios were 0.19–0.26 and 0.07–0.10. Either way, the N_e_/N_c_ ratios are similar to the mean (0.11) of 92 comprehensive empirical estimates of N_e_/N reviewed by Frankham [186]. Palstra and Fraser [188] reported a median N_e_/N of 0.23 based on 31 correctly linked N_e_/N ratios but these only included ectotherms. The main reasons for the low N_e_/N ratio in the Kruger elephants should be a fluctuating population size, variance in reproductive success and a high ratio of adult life span to age at maturity [186,190,191]. The estimated N_e_/N_c_ ratio is not unusually low [168] but it is important to note that it does not represent the current population but rather the parental population of the sampled individuals. Assuming that N_e_ did not change much in the meantime and given a present census size of 21,000 individuals, of which 8,000 are estimated to be adults [26], the current N_e_/N_c_ and N_e_/N_a_ ratios could be about 0.02–0.03 and 0.06–0.09, respectively. The long generation time of elephants may slow the rate of genetic drift but there may only be a delay in the reduction of inbreeding N_e_ and the restoration of genetic variation by mutation will be very slow [156,192]. Focusing only on current levels of genetic diversity, without an assessment of multigeneration N_e_ [193], can be misleading as an indicator of a genetically healthy population and for predicting short-term and long-term genetic risks [194]. Given the demographic collapse and recovery of the Kruger elephants over the past two centuries, a relatively brief history considering their generation time and the migration-drift disequilibrium in the GKNP’s expanding elephant population, additional future estimates of N_e_ are needed to better understand the effects on it of this complex and dynamic history [107,136,137].

## 5. Conclusions

We consider that conservation genetic assessments should be as thorough as possible, covering genetic diversity, fine- and population-scale genetic structure, genetic bottleneck testing, demographic history inferences and estimates of effective population size. Only then is it possible to obtain an accurate and detailed picture of the genetic status of populations, clues to causes of this status and a solid basis for further investigations and conservation and management plans. This study also illustrates the difficulties in determining the conservation genetic status of populations that have suffered catastrophic demographic collapse but have recovered rapidly and received gene flow, so that the genetic impacts of this complex history in a short time interval are difficult to ascertain. This is further complicated in species with long reproductive lifespan and generation time, since in these the effects on the genetic dynamics of a population of the different demographic events in the last generations may actually reflect history over the last century or more and time lags are expected to be long. In such cases, we argue that the full extent of impacts on genetic variation and N_e_ of recent dramatic changes in population size can only be understood through follow-up analyses in subsequent generations of the target population.

For reasons discussed above, such as a rapid population recovery following the demographic bottleneck during the 19th–20th century transition, immigration from neighbouring populations and a long generation time, the Kruger elephant population does not appear to have significantly lower levels of genetic diversity than other Southern African elephant populations and different methods of demographic inference also did not detect consistent signs of a recent reduction in N_e_ or deviation from mutation-drift equilibrium [28,30]. However, given their recent history the Kruger elephants are likely a nonequilibrium population, the estimates of contemporary N_e_ are at the lower end of the range of values that have been suggested as the critical minimum for the long-term maintenance of genetic variation and adaptive potential [180,181,183] and the current N_e_/N may be quite low (<0.1). Clearly, in the future there is a need for genetic monitoring [195] and analyses including samples from neighbouring populations, particularly from Mozambique and Zimbabwe, for an adequate assessment of changes and trends in N_e_ and N_e_/N, ideally also using genomic data [196]. This is even more relevant now, after the establishment of the Great Limpopo Transfrontier Park (GLTP) in 2002, with the aim of linking the KNP, LNP and Gonarezhou National Park (GNP) in Zimbabwe. With the creation of this transboundary protected area, there is an expectation of increased gene flow and local Ne and population growth to carrying capacity, as the area has an estimated mean deficit of ~70,000 elephants relative to ecological benchmarks [15], and subsequent stabilization by density-dependent and environmental factors and source-sink metapopulation dynamics [20]. However, this mega-park will only reach its promised potential for the conservation of savannah elephants if the pressures of poaching and conflict with humans are removed or at least substantially mitigated [14,197] and functional ecological corridors are available to facilitate movement and gene flow through the landscape matrix between non-adjacent constituent protected areas [198].

## Figures and Tables

**Table 1:** Estimates of genetic diversity based on 18 microsatellite loci for elephants in the APNR (*n* = 248) and KNP (*n* = 46).

	APNR						KNP					
Locus	N_A_	He	Ho	F_IS_	A_R_	PA_R_	N_A_	He	Ho	F_IS_	A_R_	PA_R_
FH1	5	0.711	0.702	0.013	4.936	0.012	5	0.712	0.717	−0.007	4.987	0.063
FH19	8	0.724	0.673	0.070	6.796	0.077	8	0.759	0.739	0.026	7.877	1.157
FH39	12	0.715	0.750	−0.049	8.261	0.847	10	0.731	0.717	0.019	9.439	2.025
FH40	6	0.512	0.548	−0.070	4.673	0.676	4	0.557	0.533	0.042	4.000	0.003
FH48	8	0.596	0.605	−0.015	6.342	0.651	7	0.639	0.652	−0.021	6.509	0.818
FH60	4	0.427	0.444	−0.039	3.113	0.153	3	0.369	0.348	0.058	2.988	0.027
FH67	8	0.666	0.625	0.062	7.006	2.035	5	0.646	0.605	0.064	4.999	0.028
FH71	3	0.564	0.500	0.114	2.998	0.000	3	0.525	0.478	0.089	3.000	0.002
FH94	6	0.700	0.671	0.042	5.034	1.034	4	0.564	0.630	−0.120	4.000	0.000
FH103	5	0.353	0.339	0.040	4.896	0.240	5	0.328	0.370	−0.127	4.758	0.102
LA5	7	0.570	0.581	−0.018	4.729	0.803	5	0.555	0.565	−0.019	4.521	0.595
LA6	4	0.463	0.492	−0.062	2.901	0.094	5	0.484	0.478	0.013	4.510	1.703
LaT06	13	0.649	0.539	0.171 *	8.590	2.715	7	0.448	0.324	0.279	6.937	1.062
LaT08	14	0.845	0.863	−0.022	11.188	0.849	13	0.861	0.870	−0.010	12.148	1.809
LaT13	11	0.800	0.742	0.073	7.520	0.933	8	0.753	0.761	−0.010	7.411	0.824
LaT18	10	0.775	0.781	−0.008	7.182	1.586	6	0.795	0.486	0.393 *	6.000	0.404
LaT24	13	0.851	0.834	0.020	9.947	2.648	8	0.827	0.658	0.206	7.916	0.617
LaT25	8	0.673	0.558	0.170 *	6.458	0.647	7	0.764	0.783	−0.025	6.746	0.935
Overall	8.06	0.644 ± 0.142	0.625 ± 0.140	0.030 *	6.25	0.89	6.28	0.629 ± 0.158	0.595 ± 0.158	0.054 *	6.04	0.68

N_A_, number of alleles; He, expected heterozygosity; Ho, observed heterozygosity; F_IS_, inbreeding coefficient; A_R_, allelic richness; PA_R_, private allelic richness. Asterisks indicate significant values after sequential Holm-Bonferroni correction with a nominal α = 0.05.

**Table 2:** Summary of the main results of spatial autocorrelation analyses using either even sample classes (with at least 100 pairwise comparisons for each distance interval) or even distance classes of 5 km.

	Even Sample Classes									Even Distance Classes (5 km)								
	D	*r*	U	L	Ur	Lr	P	ω	P_ω_	C	*r*	U	L	Ur	Lr	P	ω	P_ω_
APNR + KNP adults	2	0.019	0.013	−0.013	0.034	0.004	0.003	188.700	0.000	1663	0.004	0.006	−0.006	0.012	−0.003	0.080	224.281	0.000
	3	0.012	0.010	−0.009	0.023	0.001	0.011	115.622	0.000	-	-	-	-	-	-	-	-	-
	4	0.008	0.008	−0.007	0.017	−0.001	0.020	74.778	0.000	-	-	-	-	-	-	-	-	-
APNR + KNP adult females	3	0.040	0.024	−0.021	0.068	0.013	0.001	125.857	0.000	320	0.024	0.015	−0.013	0.042	0.007	0.001	263.182	0.000
	9	0.014	0.007	−0.007	0.025	0.003	0.000	37.493	0.000	-	-	-	-	-	-	-	-	-
APNR + KNP adult males	2	−0.001	0.020	−0.019	0.022	−0.023	0.533	180.299	0.000	604	−0.001	0.011	−0.010	0.011	−0.013	0.583	212.809	0.000
	3	0.004	0.016	−0.015	0.022	−0.013	0.290	100.978	0.002	-	-	-	-	-	-	-	-	-
APNR adults	2	0.018	0.014	−0.013	0.033	0.003	0.006	116.296	0.001	1639	0.004	0.006	−0.006	0.011	−0.003	0.122	81.399	0.002
	3	0.011	0.010	−0.009	0.022	−0.001	0.023	75.154	0.001	-	-	-	-	-	-	-	-	-
APNR adult females	3	0.038	0.023	−0.021	0.065	0.011	0.001	58.721	0.000	316	0.023	0.014	−0.013	0.040	0.005	0.002	40.909	0.004
	8	0.017	0.008	−0.007	0.029	0.005	0.000	25.058	0.001	-	-	-	-	-	-	-	-	-
	9	0.011	0.007	−0.006	0.022	0.000	0.002	23.625	0.004	-	-	-	-	-	-	-	-	-
APNR adult males	2	0.000	0.021	−0.020	0.023	−0.023	0.516	85.590	0.015	596	−0.001	0.011	−0.010	0.011	−0.013	0.573	67.208	0.028
	3	0.004	0.017	−0.015	0.022	−0.014	0.300	74.756	0.018	-	-	-	-	-	-	-	-	-

D: first distance class in km; *r*: autocorrelation coefficient; U: upper limit of the 95% CI about the null hypothesis of no spatial structure; L: lower limit of the previous 95% CI; Ur: upper limit of the 95% CI about *r*; Lr: lower limit of the 95% CI about *r*; P: *p* value; ω: value of ω metric; P_ω_: *p* value of ω test; C: number of pairwise comparisons in the first distance class. Significant *p* values are bolded.

**Table 3:** Overall and sex-specific estimates and standard deviations of genetic differentiation between the APNR and KNP elephants. Asterisks indicate significant values at α = 0.05.

	Overall	Adults	Adult Males	Adult Females
Fst	0.004 ± 0.002 *	0.004 ± 0.003 *	−0.001 ± 0.003	0.014 ± 0.007 *
G’’st	0.011 ± 0.007 *	0.010 ± 0.007 *	−0.003 ± 0.007	0.039 ± 0.018 *
Jost’s Dest	0.007 ± 0.004 *	0.006 ± 0.004 *	−0.002 ± 0.005	0.025 ± 0.012 *

**Table 4:** Estimates of N_e_ for the APNR and APNR+KNP obtained from the linkage disequilibrium (LD) method, the estimator by parentage assignments (EPA), VarEff with an average microsatellite mutation rate of 10^−3^ mutations per locus per gamete per generation (the tested rate that best fitted the data in the VarEff analyses), Migraine and the homozygosity-based estimator of *Θ* (*Θ_F_*; [112]). Values in parenthesis are 95% confidence intervals (CIs). For Migraine and *Θ_F_*, estimates are given assuming an average microsatellite mutation rate of 10^−3^ or 10^−4^ mutations per locus per gamete per generation, respectively.

	APNR	APNR+KNP
LD Method	388.3 [245.2–809.3]	510.2 [319.9–1091.4]
EPA	197 [83–436]	692 [109–743]
VarEff		
Harmonic mean	343.8	339.3
Median	454.7	443.5
90% central range	160.9–970.9	157.5–920.8
Migraine	737 [590–915]; 7370 [5900–9150]	723 [581–895]; 7230 [5810–8950]
*Θ_F_*	618.5; 6185	614.7; 6147

## Supplementary Materials

The following are available online at https://www.mdpi.com/2073-4425/10/10/779/s1. Figure S1: Plots of Mantel tests between pairwise genetic and geographic distances, Figure S2: Bayesian clustering results from separate analyses by sex of the APNR+KNP samples, Figure S3: Two-dimensional profile likelihood regions of *Θ* and *Θ*_anc_ from Migraine’s ‘OnePopVarSize’ model, Figure S4: VarEff inference of demographic history over the last 3000 generations based on the APNR+KNP data set, Table S1: Multilocus microsatellite genotypes of the 294 elephants included in this study, Table S2: Hardy-Weinberg equilibrium (HWE) test *p* values for the entire APNR dataset and for a subset of 40 unrelated individuals, Table S3: Loci suggested to have null alleles and their frequencies, as determined by different methods using the APNR dataset, Table S4: Main results of the Mantel tests of isolation by distance for all adults and for each sex separately, Table S5: Parameter estimates from the Migraine analyses using either the ‘OnePop’ model or the ‘OnePopVarSize’ model, Table S6: VarEff estimates of Ne in the last 3000 generations for the APNR, Table S7: VarEff estimates of Ne in the last 3000 generations for APNR+KNP. Table S8. Bottleneck tests for recent reduction in N_e_, Table S9: Results of *M*-ratio tests.

## References

[1] Fahrig L. (1997). Relative effects of habitat loss and fragmentation on population extinction. J. Wildl. Manag..

[2] Hoare R.E. (1999). Determinants of human-elephant conflict in a land-use mosaic. J. Appl. Ecol..

[3] Milner-Gulland E.J., Leader-Williams N. (1992). A model of incentives for the illegal exploitation of black rhinos and elephants: Poaching pays in Luangwa Valley, Zambia. J. Appl. Ecol..

[4] Winiarski J.M., Moorman C.E., Carpenter J.P., Hess G.R. (2017). Reproductive consequences of habitat fragmentation for a declining resident bird of the longleaf pine ecosystem. Ecosphere.

[5] Banks S.C., Piggott M.P., Stow A.J., Taylor A.C. (2007). Sex and sociality in a disconnected world: A review of the impacts of habitat fragmentation on animal social interactions. Can. J. Zool..

[6] Graham M.D., Douglas-Hamilton I., Adams W.M., Lee P.C. (2009). The movement of African elephants in a human-dominated land-use mosaic. Anim. Conserv..

[7] Montgomery M.E., Woodworth L.M., Nurthen R.K., Gilligan D.M., Briscoe D.A., Frankham R. (2000). Relationships between population size and loss of genetic diversity: Comparisons of experimental results with theoretical predictions. Conserv. Genet..

[8] Frankham R. (2005). Genetics and extinction. Biol. Conserv..

[9] Ouborg N.J., van Treuren R., van Damme J.M.M. (1991). The significance of genetic erosion in the process of extinction. Oecologia.

[10] Haag T., Santos A.S., Sana D.A., Morato R.G., Cullen L., Crawshaw P.G., De Angelo C., Di Bitetti M.S., Salzano F.M., Eizirik E. (2010). The effect of habitat fragmentation on the genetic structure of a top predator: Loss of diversity and high differentiation among remnant populations of Atlantic Forest jaguars (*Panthera onca*). Mol. Ecol..

[11] Stockwell C.A., Hendry A.P., Kinnison M.T. (2003). Contemporary evolution meets conservation biology. Trends Ecol. Evol..

[12] Comer C.E., Kilgo J.C., D’Angelo G.J., Glenn T.C., Miller K.V. (2005). Fine scale genetic structure and social organization in female white-tailed deer. J. Wildl. Manag..

[13] Gobush K., Kerr B., Wasser S. (2009). Genetic relatedness and disrupted social structure in a poached population of African elephants. Mol. Ecol..

[14] Chase M.J., Schlossberg S., Griffin C.R., Bouché P.J.C., Djene S.W., Elkan P.W., Ferreira S., Grossman F., Kohi E.M., Landen K. (2016). Continent-wide survey reveals massive decline in African savannah elephants. PeerJ.

[15] Robson A.S., Trimble M.J., Purdon A., Young-Overton K.D., Pimm S.L., van Aarde R.J. (2017). Savanna elephant numbers are only a quarter of their expected values. PLoS ONE.

[16] Blanc J. (2008). Loxodonta Africana. The IUCN Red List of Threatened Species 2008: E.T12392A3339343.

[17] Archie E.A., Chiyo P.I. (2012). Elephant behaviour and conservation: Social relationships, the effects of poaching, and genetic tools for management. Mol. Ecol..

[18] Douglas-Hamilton I. (1987). African elephants: Population trends and their causes. Oryx.

[19] Milner-Gulland E.J., Mace R. (1991). The impact of the ivory trade on the African elephant *Loxodonta africana* population as assessed by data from the trade. Biol. Conserv..

[20] van Aarde R., Jackson T. (2007). Megaparks for metapopulations: Addressing the causes of locally high elephant numbers in southern Africa. Biol. Conserv..

[21] Nyakaana S., Arctander P., Siegismund H.R. (2002). Population structure of the African savannah elephant inferred from mitochondrial control region sequences and nuclear microsatellite loci. Heredity.

[22] Comstock K.E., Georgiadis N., Pecon-Slattery J., Roca A.L., Ostrander E.A., O’Brien S.J., Wasser S.K. (2002). Patterns of molecular genetic variation among African elephant populations. Mol. Ecol..

[23] Roca A.L. (2001). Genetic evidence for two species of elephant in Africa. Science.

[24] Roca A.L., Georgiadis N., O’Brien S.J. (2005). Cytonuclear genomic dissociation in African elephant species. Nat. Genet..

[25] Hall-Martin A.J. (1992). Distribution and status of the African elephant *Loxodonta africana* in South Africa, 1652–1992. Koedoe.

[26] Selier S.A.J., Henley M., Pretorius Y., Garai M., Child M.F., Roxburgh L., Do Linh San E., Raimondo D., Davies-Mostert H.T. (2016). A conservation assessment of *Loxodonta africana*. The Red List of Mammals of South Africa, Swaziland and Lesotho.

[27] Lacy R.C. (1997). Importance of genetic variation to the viability of mammalian populations. J. Mammal..

[28] Whitehouse A.M., Harley E.H. (2001). Post-bottleneck genetic diversity of elephant populations in South Africa, revealed using microsatellite analysis. Mol. Ecol..

[29] DeSalle R., Amato G. (2004). The expansion of conservation genetics. Nat. Rev. Genet..

[30] de Flamingh A., Roca A.L., van Aarde R.J. (2018). Origin and phylogeography of African savannah elephants (*Loxodonta africana*) in Kruger and nearby parks in southern Africa. Conserv. Genet..

[31] Whyte I., Aarde R., Pimm S.L. (1998). Managing the elephants of Kruger National Park. Anim. Conserv..

[32] Henley M.D. (2014). Report on Elephant Movements in Relation to Water and the Effect of the 2012 Floods within the Associated Private Nature Reserves.

[33] Moss C.J. (2001). The demography of an African elephant (*Loxodonta africana*) population in Amboseli, Kenya. J. Zool..

[34] Henley M. (2013). Report on the Demographics of the Bull Population of the Associated Private Nature Reserves.

[35] Comstock K.E., Wasser S.K., Ostrander E.A. (2000). Polymorphic microsatellite DNA loci identified in the African elephant (*Loxodonta africana*). Mol. Ecol..

[36] Eggert L.S., Ramakrishnan U., Mundy N.I., Woodruff D.S. (2000). Polymorphic microsatellite DNA markers in the African elephant (*Loxondonta africana*) and their use in the Asian elephant (*Elephas maximus)*. Mol. Ecol..

[37] Archie E.A., Moss C.J., Alberts S.C. (2003). Characterization of tetranucleotide microsatellite loci in the African Savannah Elephant (*Loxodonta africana africana*). Mol. Ecol. Notes.

[38] Ahlering M.A., Hailer F., Roberts M.T., Foley C. (2011). A simple and accurate method to sex savannah, forest and Asian elephants using noninvasive sampling techniques. Mol. Ecol. Resour..

[39] Frantz A.C., Pope L.C., Carpenter P.J., Roper T.J., Wilson G.J., Delahay R.J., Burke T. (2003). Reliable microsatellite genotyping of the Eurasian badger (*Meles meles*) using faecal DNA. Mol. Ecol..

[40] Hansen H., Ben-David M., Mcdonald D.B. (2008). TECHNICAL ADVANCES: Effects of genotyping protocols on success and errors in identifying individual river otters (*Lontra canadensis*) from their faeces. Mol. Ecol. Resour..

[41] Valière N. (2002). GIMLET: A computer program for analysing genetic individual identification data. Mol. Ecol. Resour..

[42] Broquet T., Petit E. (2004). Quantifying genotyping errors in noninvasive population genetics. Mol. Ecol..

[43] Waits L.P., Luikart G., Taberlet P. (2001). Estimating the probability of identity among genotypes in natural populations: Cautions and guidelines. Mol. Ecol..

[44] Kalinowski S.T., Taper M.L., Marshall T.C. (2007). Revising how the computer program cervus accommodates genotyping error increases success in paternity assignment. Mol. Ecol..

[45] Kalinowski S.T., Sawaya M.A., Taper M.L. (2006). Individual identification and distribution of genotypic differences between individuals. J. Wildl. Manag..

[46] Van Oosterhout C., Hutchinson W.F., Wills D.P.M., Shipley P. (2004). MICRO-CHECKER: Software for identifying and correcting genotyping errors in microsatellite data. Mol. Ecol. Notes.

[47] Rousset F. (2008). Genepop’007: A complete re-implementation of the genepop software for Windows and Linux. Mol. Ecol. Resour..

[48] Holm S. (1979). A simple sequentially rejective multiple test procedure. Scand. J. Stat..

[49] Gaetano J. Holm-Bonferroni Sequential Correction: An EXCEL Calculator (1.2) [Microsoft Excel Workbook]. https://www.researchgate.net/publication/242331583_Holm-Bonferroni_Sequential_Correction_An_EXCEL_Calculator_-_Ver_12.

[50] Dąbrowski M.J., Bornelöv S., Kruczyk M., Baltzer N., Komorowski J. (2015). ‘True’ null allele detection in microsatellite loci: A comparison of methods, assessment of difficulties and survey of possible improvements. Mol. Ecol. Resour..

[51] Summers K., Amos W. (1997). Behavioral, ecological, and molecular genetic analyses of reproductive strategies in the Amazonian dart-poison frog, *Dendrobates ventrimaculatus*. Behav. Ecol..

[52] Kalinowski S.T., Taper M.L. (2006). Maximum likelihood estimation of the frequency of null alleles at microsatellite loci. Conserv. Genet..

[53] Chybicki I.J., Burczyk J. (2009). Simultaneous estimation of null alleles and inbreeding coefficients. J. Hered..

[54] Kalinowski S.T. (2005). hp-rare 1.0: A computer program for performing rarefaction on measures of allelic richness. Mol. Ecol. Notes.

[55] Belkhir K., Borsa P., Chikhi L., Bonhomme F. (1996). Genetix 4.05: Windows^TM^ Software for Population Genetics.

[56] Whyte I. (2001). Conservation management of the Kruger National Park elephant population. Ph.D. Thesis.

[57] Archie E.A., Maldonado J.E., Hollister-Smith J.A., Poole J.H., Moss C.J., Fleischer R.C., Alberts S.C. (2008). Fine-scale population genetic structure in a fission-fusion society. Mol. Ecol..

[58] Kioko J., Muruthi P., Omondi P., Chiyo P.I. (2008). The performance of electric fences as elephant barriers in Amboseli, Kenya. S. Afr. J. Wildl. Res..

[59] Loarie S.R., Aarde R.J.V., Pimm S.L. (2009). Fences and artificial water affect African savannah elephant movement patterns. Biol. Conserv..

[60] Osborn F.V., Parker G.E. (2003). Towards an integrated approach for reducing the conflict between elephants and people: A review of current research. Oryx.

[61] Druce H.C., Pretorius K., Slotow R. (2008). The response of an elephant population to conservation area expansion: Phinda Private Game Reserve, South Africa. Biol. Conserv..

[62] Harris G.M., Russell G.J., van Aarde R.I., Pimm S.L. (2008). Rules of habitat use by elephants *Loxodonta africana* in southern Africa: Insights for regional management. Oryx.

[63] Peakall R., Smouse P.E. (2006). GenAlEx 6: Genetic analysis in Excel. Population genetic software for teaching and research. Mol. Ecol. Notes.

[64] Peakall R., Smouse P.E. (2012). GenAlEx 6.5: Genetic analysis in Excel. Population genetic software for teaching and research—An update. Bioinformatics.

[65] Smouse P.E., Peakall R. (1999). Spatial autocorrelation analysis of individual multiallele and multilocus genetic structure. Heredity.

[66] Moran P.A.P. (1950). Notes on continuous stochastic phenomena. Biometrika.

[67] Peakall R., Ruibal M., Lindenmayer D.B. (2003). Spatial autocorrelation analysis offers insights into gene flow in the Australian bush rat, *Rattus fuscipes*. Evolution.

[68] Epperson B.K. (2005). Estimating dispersal from short distance spatial autocorrelation. Heredity.

[69] Dubey S., Brown G.P., Madsen T., Shine R. (2008). Male-biased dispersal in a tropical Australian snake (*Stegonotus cucullatus*, *Colubridae*). Mol. Ecol..

[70] Smouse P.E., Peakall R., Gonzales E. (2008). A heterogeneity test for fine-scale genetic structure. Mol. Ecol..

[71] Banks S.C., Peakall R. (2012). Genetic spatial autocorrelation can readily detect sex-biased dispersal. Mol. Ecol..

[72] Mantel N. (1967). The detection of disease clustering and a generalized regression approach. Cancer Res..

[73] Frantz A.C., Hamann J.-L., Klein F. (2008). Fine-scale genetic structure of red deer (*Cervus elaphus*) in a French temperate forest. Eur. J. Wildl. Res..

[74] Rousset F. (2000). Genetic differentiation between individuals. J. Evol. Biol..

[75] Loiselle B.A., Sork V.L., Nason J., Graham C. (1995). Spatial genetic structure of a tropical understory shrub, *Psychotria officinalis* (Rubiaceae). Am. J. Bot..

[76] Hardy O.J., Vekemans X. (2002). SPAGeDi: A versatile computer program to analyse spatial genetic structure at the individual or population levels. Mol. Ecol. Resour..

[77] Pritchard J.K., Stephens M., Donnelly P. (2000). Inference of population structure using multilocus genotype data. Genetics.

[78] Guillot G., Mortier F., Estoup A. (2005). GENELAND: A computer package for landscape genetics. Mol. Ecol. Resour..

[79] Chen C., Durand E., Forbes F., François O. (2007). Bayesian clustering algorithms ascertaining spatial population structure: A new computer program and a comparison study. Mol. Ecol. Notes.

[80] Blair C., Weigel D.E., Balazik M., Keeley A.T.H., Walker F.M., Landguth E., Cushman S., Murphy M., Waits L., Balkenhol N. (2012). A simulation-based evaluation of methods for inferring linear barriers to gene flow. Mol. Ecol. Resour..

[81] Latch E.K., Dharmarajan G., Glaubitz J.C., Rhodes O.E. (2006). Relative performance of Bayesian clustering software for inferring population substructure and individual assignment at low levels of population differentiation. Conserv. Genet..

[82] François O., Durand E. (2010). Spatially explicit Bayesian clustering models in population genetics. Mol. Ecol. Resour..

[83] Basto M.P., Santos-Reis M., Simões L., Grilo C., Cardoso L., Cortes H., Bruford M.W., Fernandes C. (2016). Assessing genetic structure in common but ecologically distinct carnivores: The stone marten and red fox. PLoS ONE.

[84] Blanchet E., Lecoq M., Sword G.A., Berthier K., Pages C., Billot C., Rivallan R., Foucart A., Vassal J.-M., Risterucci A.M. (2012). A comparative analysis of fine-scale genetic structure in three closely related syntopic species of the grasshopper genus *Calliptamus*. Can. J. Zool..

[85] Guillot G. (2008). Inference of structure in subdivided populations at low levels of genetic differentiation—The correlated allele frequencies model revisited. Bioinformatics.

[86] Earl D.A., vonHoldt B.M. (2012). STRUCTURE HARVESTER: A website and program for visualizing STRUCTURE output and implementing the Evanno method. Conserv. Genet. Resour..

[87] Jakobsson M., Rosenberg N.A. (2007). CLUMPP: A cluster matching and permutation program for dealing with label switching and multimodality in analysis of population structure. Bioinformatics.

[88] Rosenberg N.A. (2003). Distruct: A program for the graphical display of population structure. Mol. Ecol. Notes.

[89] Goudet J., Raymond M., de Meeus T., Rousset F. (1996). Testing differentiation in diploid populations. Genetics.

[90] Goudet J. (1995). FSTAT (version 1.2): A computer program to calculate F-statistics. J. Hered..

[91] Weir B.S., Cockerham C.C. (1984). Estimating F-statistics for the analysis of population structure. Evolution.

[92] Meirmans P.G., Hedrick P.W. (2011). Assessing population structure: FST and related measures. Mol. Ecol. Resour..

[93] Jost L. (2008). Gst and its relatives do not measure differentiation. Mol. Ecol..

[94] Meirmans P.G., Van Tienderen P.H. (2004). Genotype and genodive: Two programs for the analysis of genetic diversity of asexual organisms. Mol. Ecol. Notes.

[95] Goudet J., Perrin N., Waser P. (2002). Tests for sex-biased dispersal using bi-parentally inherited genetic markers. Mol. Ecol..

[96] Wright S. (1931). Evolution in Mendelian populations. Genetics.

[97] Waples R.S. (2006). A bias correction for estimates of effective population size based on linkage disequilibrium at unlinked gene loci. Conserv. Genet..

[98] Do C., Waples R.S., Peel D., Macbeth G.M., Tillett B.J., Ovenden J.R. (2014). NeEstimator v2: Re-implementation of software for the estimation of contemporary effective population size Ne from genetic data. Mol. Ecol. Resour..

[99] Peel D., Waples R.S., Macbeth G.M., Do C., Ovenden J.R. (2013). Accounting for missing data in the estimation of contemporary genetic effective population size (Ne). Mol. Ecol. Resour..

[100] Waples R.S., Do C. (2010). Linkage disequilibrium estimates of contemporary Ne using highly variable genetic markers: A largely untapped resource for applied conservation and evolution. Evol. Appl..

[101] Jones A.T., Ovenden J.R., Wang Y.-G. (2016). Improved confidence intervals for the linkage disequilibrium method for estimating effective population size. Heredity.

[102] Gilbert K.J., Whitlock M.C. (2015). Evaluating methods for estimating local effective population size with and without migration. Evolution.

[103] Waples R.S., England P.R. (2011). Estimating contemporary effective population size on the basis of linkage disequilibrium in the face of migration. Genetics.

[104] Wang J. (2016). A comparison of single-sample estimators of effective population sizes from genetic marker data. Mol. Ecol..

[105] Sved J.A., Cameron E.C., Gilchrist A.S. (2013). Estimating effective population size from linkage disequilibrium between unlinked loci: Theory and application to fruit fly outbreak populations. PLoS ONE.

[106] Holleley C.E., Nichols R.A., Whitehead M.R., Adamack A.T., Gunn M.R., Sherwin W.B. (2014). Testing single-sample estimators of effective population size in genetically structured populations. Conserv. Genet..

[107] Luikart G., Ryman N., Tallmon D.A., Schwartz M.K., Allendorf F.W. (2010). Estimation of census and effective population sizes: The increasing usefulness of DNA-based approaches. Conserv. Genet..

[108] Robinson J.D., Moyer G.R. (2013). Linkage disequilibrium and effective population size when generations overlap. Evol. Appl..

[109] Waples R.S., Antao T., Luikart G. (2014). Effects of overlapping generations on linkage disequilibrium estimates of effective population size. Genetics.

[110] Sinnock P. (1975). The Wahlund Effect for the Two-Locus Model. Am. Nat..

[111] Wang J., Brekke P., Huchard E., Knapp L.A., Cowlishaw G. (2010). Estimation of parameters of inbreeding and genetic drift in populations with overlapping generations. Evolution.

[112] Xu H., Fu Y.-X. (2004). Estimating effective population size or mutation rate with microsatellites. Genetics.

[113] Ellegren H. (2004). Microsatellites: Simple sequences with complex evolution. Nat. Rev. Genet..

[114] Weber J.L., Wong C. (1993). Mutation of human short tandem repeats. Hum. Mol. Genet..

[115] Whittaker J.C., Harbord R.M., Boxall N., Mackay I., Dawson G., Sibly R.M. (2003). Likelihood-based estimation of microsatellite mutation rates. Genetics.

[116] Xu X., Peng M., Fang Z., Xu X. (2000). The direction of microsatellite mutations is dependent upon allele length. Nat. Genet..

[117] Piry S., Luikart G., Cornuet J.-M. (1999). BOTTLENECK: A computer program for detecting recent reductions in the effective size using allele frequency data. J. Hered..

[118] Cornuet J.M., Luikart G. (1996). Description and power analysis of two tests for detecting recent population bottlenecks from allele frequency data. Genetics.

[119] Luikart G., Cornuet J.-M. (1998). Empirical evaluation of a test for identifying recently bottlenecked populations from allele frequency. Conserv. Biol..

[120] Peery M.Z., Kirby R., Reid B.N., Stoelting R., Doucet-BëEr E., Robinson S., VáSquez-Carrillo C., Pauli J.N., PalsbøLl P.J. (2012). Reliability of genetic bottleneck tests for detecting recent population declines. Mol. Ecol..

[121] Luikart G., Allendorf F.W., Cornuet J.M., Sherwin W.B. (1998). Distortion of allele frequency distributions provides a test for recent population bottlenecks. J. Hered..

[122] Garza J.C., Williamson E.G. (2001). Detection of reduction in population size using data from microsatellite loci. Mol. Ecol..

[123] Leblois R., Pudlo P., Néron J., Bertaux F., Reddy Beeravolu C., Vitalis R., Rousset F. (2014). Maximum-likelihood inference of population size contractions from microsatellite data. Mol. Biol. Evol..

[124] Beaumont M.A. (1999). Detecting population expansion and decline using microsatellites. Genetics.

[125] Faurby S., Pertoldi C. (2012). The consequences of the unlikely but critical assumption of stepwise mutation in the population genetic software, MSVAR. Evol. Ecol. Res..

[126] Girod C., Vitalis R., Leblois R., Fréville H. (2011). Inferring population decline and expansion from microsatellite data: A simulation-based evaluation of the Msvar method. Genetics.

[127] Nikolic N., Chevalet C. (2014). Detecting past changes of effective population size. Evol. Appl..

[128] Pompanon F., Bonin A., Bellemain E., Taberlet P. (2005). Genotyping errors: Causes, consequences and solutions. Nat. Rev. Genet..

[129] McKelvey K.S., Schwartz M.K. (2004). Genetic errors associated with population estimation using non-invasive molecular tagging: Problems and new solutions. J. Wildl. Manag..

[130] Carlsson J. (2008). Effects of microsatellite null alleles on assignment testing. J. Hered..

[131] Chapuis M.-P., Estoup A. (2007). Microsatellite null alleles and estimation of population differentiation. Mol. Biol. Evol..

[132] Dakin E.E., Avise J.C. (2004). Microsatellite null alleles in parentage analysis. Heredity.

[133] Huang K., Ritland K., Dunn D.W., Qi X., Guo S., Li B. (2016). Estimating relatedness in the presence of null alleles. Genetics.

[134] Storz J.F., Ramakrishnan U., Alberts S.C. (2002). Genetic effective size of a wild primate population: Influence of current and historical demography. Evolution.

[135] Waples R.S. (2015). Testing for Hardy–Weinberg Proportions: Have we lost the plot?. J. Hered..

[136] Kamath P.L., Haroldson M.A., Luikart G., Paetkau D., Whitman C., van Manen F.T. (2015). Multiple estimates of effective population size for monitoring a long-lived vertebrate: An application to Yellowstone grizzly bears. Mol. Ecol..

[137] Skrbinšek T., Jelenčič M., Waits L., Kos I., Jerina K., Trontelj P. (2012). Monitoring the effective population size of a brown bear (*Ursus arctos*) population using new single-sample approaches. Mol. Ecol..

[138] Neel M.C., McKelvey K., Ryman N., Lloyd M.W., Short Bull R., Allendorf F.W., Schwartz M.K., Waples R.S. (2013). Estimation of effective population size in continuously distributed populations: There goes the neighborhood. Heredity.

[139] Dobson F.S., Chesser R.K., Hoogland J.L., Sugg D.W., Foltz D.W. (2004). The influence of social breeding groups on effective population size in black-tailed prairie dogs. J. Mammal..

[140] Wang J. (1997). Effective size and F-statistics of subdivided populations. Genetics.

[141] Miller M.P., Mullins T.D., Haig S.M., Takano L., Garcia K. (2015). Genetic structure, diversity, and interisland dispersal in the endangered Mariana Common Moorhen (*Gallinula chloropus guami*). Condor.

[142] Leblois R. (2018). Personal communication.

[143] Valdes A.M., Slatkin M., Freimert N.B. (1993). Allele frequencies at microsatellite loci: The stepwise mutation model revisited. Genetics.

[144] Di Rienzo A., Peterson A.C., Garza J.C., Valdes A.M., Slatkin M., Freimer N.B. (1994). Mutational processes of simple-sequence repeat loci in human populations. Proc. Natl. Acad. Sci. USA.

[145] Ishida Y., Van Coeverden de Groot P.J., Leggett K.E.A., Putnam A.S., Fox V.E., Lai J., Boag P.T., Georgiadis N.J., Roca A.L. (2016). Genetic connectivity across marginal habitats: The elephants of the Namib Desert. Ecol. Evol..

[146] Ishengoma D.R.S., Shedlock A.M., Foley C.A.H., Foley L.J., Wasser S.K., Balthazary S.T., Mutayoba B.M. (2008). Effects of poaching on bull mating success in a free ranging African elephant (*Loxodonta africana*) population in Tarangire National Park, Tanzania. Conserv. Genet..

[147] Ahlering M.A., Eggert L.S., Western D., Estes A., Munishi L., Fleischer R., Roberts M., Maldonado J.E. (2012). Identifying source populations and genetic structure for savannah elephants in human-dominated landscapes and protected areas in the Kenya-Tanzania borderlands. PLoS ONE.

[148] Okello J.B.A., Masembe C., Rasmussen H.B., Wittemyer G., Omondi P., Kahindi O., Muwanika V.B., Arctander P., Douglas-Hamilton I., Nyakaana S. (2008). Population genetic structure of savannah elephants in Kenya: Conservation and management implications. J. Hered..

[149] Okello J.B.A., Wittemyer G., Rasmussen H.B., Arctander P., Nyakaana S., Douglas-Hamilton I., Siegismund H.R. (2008). Effective population size dynamics reveal impacts of historic climatic events and recent anthropogenic pressure in African elephants. Mol. Ecol..

[150] Spencer C.C., Neigel J.E., Leberg P.L. (2000). Experimental evaluation of the usefulness of microsatellite DNA for detecting demographic bottlenecks. Mol. Ecol..

[151] Nyakaana S., Arctander P. (1999). Population genetic structure of the African elephant in Uganda based on variation at mitochondrial and nuclear loci: Evidence for male-biased gene flow. Mol. Ecol..

[152] Williamson-Natesan E.G. (2005). Comparison of methods for detecting bottlenecks from microsatellite loci. Conserv. Genet..

[153] Hundertmark K.J., Van Daele L.J. (2010). Founder effect and bottleneck signatures in an introduced, insular population of elk. Conserv. Genet..

[154] Hoban S.M., Gaggiotti O.E., Bertorelle G. (2013). The number of markers and samples needed for detecting bottlenecks under realistic scenarios, with and without recovery: A simulation-based study. Mol. Ecol..

[155] Busch J.D., Waser P.M., DeWoody J.A. (2007). Recent demographic bottlenecks are not accompanied by a genetic signature in banner-tailed kangaroo rats (*Dipodomys spectabilis*). Mol. Ecol..

[156] Lippé C., Dumont P., Bernatchez L. (2006). High genetic diversity and no inbreeding in the endangered copper redhorse, *Moxostoma hubbsi* (Catostomidae, Pisces): The positive sides of a long generation time. Mol. Ecol..

[157] Waples R.S., Gaggiotti O. (2006). What is a population? An empirical evaluation of some genetic methods for identifying the number of gene pools and their degree of connectivity. Mol. Ecol..

[158] Temple H.J., Hoffman J.I., Amos W. (2006). Dispersal, philopatry and intergroup relatedness: Fine-scale genetic structure in the white-breasted thrasher, *Ramphocinclus brachyurus*. Mol. Ecol..

[159] Hu Y., Zhan X., Qi D., Wei F. (2010). Spatial genetic structure and dispersal of giant pandas on a mountain-range scale. Conserv. Genet..

[160] De Villiers P.A., Kok O.B. (1997). Home range, association and related aspects of elephants in the eastern Transvaal Lowveld. Afr. J. Ecol..

[161] Ahlering M.A., Maldonado J.E., Fleischer R.C., Western D., Eggert L.S. (2012). Fine-scale group structure and demography of African savanna elephants recolonizing lands outside protected areas. Divers. Distrib..

[162] Wakeley J. (1999). Nonequilibrium migration in human history. Genetics.

[163] Chikhi L., Sousa V.C., Luisi P., Goossens B., Beaumont M.A. (2010). The confounding effects of population structure, genetic diversity and the sampling scheme on the detection and quantification of population size changes. Genetics.

[164] Charlesworth B. (2009). Fundamental concepts in genetics: Effective population size and patterns of molecular evolution and variation. Nat. Rev. Genet..

[165] Nunney L., Elam D.R. (1994). Estimating the effective population size of conserved populations. Conserv. Biol..

[166] Leberg P. (2005). Genetic approaches for estimating the effective size of populations. J. Wildl. Manag..

[167] Nunney L., Campbell K.A. (1993). Assessing minimum viable population size: Demography meets population genetics. Trends Ecol. Evol..

[168] Palstra F.P., Ruzzante D.E. (2008). Genetic estimates of contemporary effective population size: What can they tell us about the importance of genetic stochasticity for wild population persistence?. Mol. Ecol..

[169] Waples R.S. (2005). Genetic estimates of contemporary effective population size: To what time periods do the estimates apply?. Mol. Ecol..

[170] Waples R.S., Do C., Chopelet J. (2011). Calculating Ne and Ne/N in age-structured populations: A hybrid Felsenstein-Hill approach. Ecology.

[171] Nei M., Li W.-H. (1973). Linkage disequilibrium in subdivided populations. Genetics.

[172] Waples R.S. (2016). Making sense of genetic estimates of effective population size. Mol. Ecol..

[173] Sun J.X., Helgason A., Masson G., Ebenesersdóttir S.S., Li H., Mallick S., Gnerre S., Patterson N., Kong A., Reich D. (2012). A direct characterization of human mutation based on microsatellites. Nat. Genet..

[174] Yang J., Jiang Z. (2011). Genetic diversity, population genetic structure and demographic history of Przewalski’s gazelle (*Procapra przewalskii*): Implications for conservation. Conserv. Genet..

[175] Waples R.S. (2010). Spatial-temporal stratifications in natural populations and how they affect understanding and estimation of effective population size. Mol. Ecol. Resour..

[176] Lehmann T., Hawley W.A., Grebert H., Collins F.H. (1998). The effective population size of Anopheles gambiae in Kenya: Implications for population structure. Mol. Biol. Evol..

[177] Beerli P., Felsenstein J. (2001). Maximum likelihood estimation of a migration matrix and effective population sizes in n subpopulations by using a coalescent approach. Proc. Natl. Acad. Sci. USA.

[178] Vitalis R., Couvet D. (2001). Estimation of effective population size and migration rate from one- and two-locus identity measures. Genetics.

[179] Franklin I.R. (2014). The 50/500 rule is still valid—Reply to Frankham et al. Biol. Conserv..

[180] Franklin I.R., Frankham R. (1998). How large must populations be to retain evolutionary potential?. Anim. Conserv..

[181] Lynch M., Lande R. (1998). The critical effective size for a genetically secure population. Anim. Conserv..

[182] Frankham R., Bradshaw C.J.A., Brook B.W. (2014). 50/500 rules need upward revision to 100/1000—Response to Franklin et al. Biol. Conserv..

[183] Frankham R., Bradshaw C.J.A., Brook B.W. (2014). Genetics in conservation management: Revised recommendations for the 50/500 rules, Red List criteria and population viability analyses. Biol. Conserv..

[184] Rowe G., Beebee T.J.C. (2004). Reconciling genetic and demographic estimators of effective population size in the anuran amphibian *Bufo calamita*. Conserv. Genet..

[185] Nunney L. (1995). Measuring the ratio of effective population size to adult numbers using genetic and ecological data. Evolution.

[186] Frankham R. (1995). Effective population size/adult population size ratios in wildlife: A review. Genet. Res..

[187] Gaggiotti O.E. (2003). Genetic threats to population persistence. Ann. Zool. Fenn..

[188] Palstra F.P., Fraser D.J. (2012). Effective/census population size ratio estimation: A compendium and appraisal. Ecol. Evol..

[189] Vucetich J.A., Waite T.A., Nunney L. (1997). Fluctuating population size and the ratio of effective to census population size. Evolution.

[190] Waite T.A., Parker P.G. (1996). Dimensionless life histories and effective population size. Conserv. Biol..

[191] Waples R.S., Luikart G., Faulkner J.R., Tallmon D.A. (2013). Simple life-history traits explain key effective population size ratios across diverse taxa. Proc. R. Soc. B Biol. Sci..

[192] Kuo C.-H., Janzen F.J. (2004). Genetic effects of a persistent bottleneck on a natural population of ornate box turtles (*Terrapene ornata*). Conserv. Genet..

[193] Kalinowski S.T., Waples R.S. (2002). Relationship of effective to census size in fluctuating populations. Conserv. Biol..

[194] Bishop J.M., Leslie A.J., Bourquin S.L., O’Ryan C. (2009). Reduced effective population size in an overexploited population of the Nile crocodile (*Crocodylus niloticus*). Biol. Conserv..

[195] Schwartz M., Luikart G., Waples R. (2007). Genetic monitoring as a promising tool for conservation and management. Trends Ecol. Evol..

[196] Nunziata S.O., Weisrock D.W. (2018). Estimation of contemporary effective population size and population declines using RAD sequence data. Heredity.

[197] Cook R.M., Henley M.D., Parrini F. (2015). Elephant movement patterns in relation to human inhabitants in and around the Great Limpopo Transfrontier Park. Koedoe.

[198] Roever C.L., van Aarde R.J., Leggett K. (2013). Functional connectivity within conservation networks: Delineating corridors for African elephants. Biol. Conserv..

