# Supplementary material for: Conservation Genetic Assessment of Savannah Elephants (Loxodonta africana) in the Greater Kruger Biosphere, South Africa
Source: Genes (Basel). 2019 Oct 5;10(10):779. doi: 10.3390/genes10100779 (PMC6826889; doi:10.3390/genes10100779)
Supplement: Supplementary file 1 [file genes-10-00779-s001.pdf]

Conservation Genetic Assessment of Savannah Elephants (*Loxodonta africana*) in the Greater Kruger Biosphere, South Africa

Teresa L. Santos (1,2,3)*, Carlos Fernandes (3)*, Michelle D. Henley (4, 5), Deborah A. Dawson (2), Hannah S. Mumby (1,6,7,8)†

1. Bull Elephant Network Project, Conservation Science Group, David Attenborough Building, Pembroke St, Cambridge, CB2 3QY, United Kingdom
2. NERC Biomolecular Analysis Facility, Department of Animal and Plant Sciences, University of Sheffield, Western Bank, Sheffield, South Yorkshire, S10 2TN, United Kingdom
3. cE3c – Centre for Ecology, Evolution and Environmental Changes, Departamento de Biologia Animal, Faculdade de Ciências, Universidade de Lisboa, 1749-016 Lisboa, Portugal
4. Applied Behavioural Ecology and Ecosystem Research Unit, University of South Africa, Florida Campus, Johannesburg, South Africa
5. Elephants Alive, Hoedspruit, South Africa
6. Centre for African Ecology, School of Animal, Plant and Environmental Sciences, University of Witwatersrand, Johannesburg, South Africa
7. Wissenschaftskolleg zu Berlin, Wallotstraße 19, Berlin, Germany
8. School of Biological Sciences and Department of Politics and Public Administration, University of Hong Kong, Hong Kong

† Corresponding author: H. S. Mumby

* T. Santos and C. Fernandes contributed equally to this work.

Index

Supplementary Methods 3

Laboratory procedures and microsatellite quality control 3

Supplementary Tables 6

Supplementary Table 1. Multilocus microsatellite genotypes 6

Supplementary Table 2. Hardy-Weinberg equilibrium (HWE) test 6

Supplementary Table 3. Loci suggested to have null alleles, and their frequencies 7

Supplementary Table 4. Main results of the Mantel tests of isolation by distance 7

Supplementary Table 5. Parameter estimates from the Migraine analyses 8

Supplementary Table 6. VarEff estimates of Ne in the last 3,000 generations for the APNR 8

Supplementary Table 7. VarEff estimates of Ne in the last 3,000 generations for APNR + KNP.. 9

Supplementary Table 8. Bottleneck tests for recent reduction in Ne 10

Supplementary Table 9. Results of M-ratio tests 10

Supplementary Figures 11

Supplementary Figure 1. Plots of Mantel tests 11

Supplementary Figure 2. Bayesian clustering results 12

Supplementary Figure 3. Two-dimensional profile likelihood regions of Θ and Θanc from Migraine's 'OnePopVarSize' model 14

Supplementary Figure 4. VarEff inference of demographic history over the last 3,000 generations 15

Supplementary Information

Supplementary Methods

Laboratory procedures and microsatellite quality control

DNA was extracted from the samples stored in ethanol or salt solution using the QIAamp Fast DNA Stool Mini Kit (QIAGEN). Extractions followed the manufacturer's protocol with the following modifications: all centrifugation steps were performed at 13,000 rpm, 2 ml microcentrifuge tubes were used instead of 1.5 ml tubes, and the DNA was eluted in two stages using a total of 100 µl of elution buffer for samples in salt solution or using a total of 50 µl for ethanol-preserved samples. The choice of the different elution volumes was based on the average DNA concentration obtained in pilot extraction tests on samples, including replicates of the same dung, stored in each of the two preservation solutions. The quality of the DNA was assessed by electrophoresis of 2 µl of extract in a 1% agarose gel with ethidium bromide. The concentration of DNA in each extract was quantified by fluorescence using the FLUOstar OPTIMA microplate reader (BMG Labtech, Germany), and diluted with 'Low TE' buffer to a concentration of approximately 20 ng/µl. After preliminary optimization of PCR conditions and multiplex combinations, the samples were genotyped for 18 species-specific autosomal microsatellite loci: FH1, FH19, FH39, FH40, FH48, FH60, FH67, FH71, FH94, FH103 [1], LA5, LA6 [2], LaT06, LaT08, LaT13, LaT18, LaT24, and LaT25 [3]. These markers were chosen because they were previously used to genotype the 46 KNP samples provided by Samuel Wasser's team, thus allowing APNR-KNP comparisons. A 'PIG-tail' sequence (GTGTCTT) was added to the 5' end of reverse primers to minimise variability in non-templated adenylation of amplicons [4]. The markers were divided into three multiplex sets (multiplex 1: FH48, FH67, FH94, FH103, LA5, LA6, LaT06, LaT13, LaT24; multiplex 2: FH19, FH39, FH40, FH60, FH71, LaT08; multiplex 3: FH1, LaT18, LaT25). Each PCR reaction contained 1 µl of multiplex primer mix (fluorescently labelled forward primer and unlabelled reverse primer) with each primer at 0.2 µM, 1 µl of QIAGEN Multiplex PCR Kit (QIAGEN, Germany), and either 1 µl or 2 µl of DNA. 1 µl was used if the DNA concentration in the sample ranged between 10 and 20 ng/µl and 2 µl if the concentration was below 10 ng/µl. PCR amplification was carried out using a DNA Engine Tetrad Thermal Cycler (MJ Research). The PCR protocol consisted of an initial denaturation at 94 °C for 15 min, followed by 45 cycles of 30 s at 94 °C, 90 s at 56 °C and 90 s at 72 °C and a final extension at 72 °C for 10 min. Negative controls were included in DNA extractions and PCR amplifications to monitor for contamination. PCR products were diluted 1:800 with double-distilled water and resolved using a 3730 DNA Analyser (Applied Biosystems, USA). Allele scoring was done using GeneMapper 3.7 (Applied Biosystems). To verify the genotypes, all samples were initially genotyped twice for each locus. When the two replicates yielded a homozygous result, an unclear heterozygote, or inconsistent genotypes, we performed additional replicates until matching heterozygotes were scored at least twice and matching homozygotes at least three times [5,6]. The samples were also genotyped for three sexing markers, PLP1, SRY1 and AMELY2 [7].

For each sample, the replicate single-locus microsatellite genotypes were compared both manually and using Gimlet 1.3.3 [8], the latter being used to construct consensus genotypes. Single-locus consensus genotypes were created using only alleles that were observed at least twice. Gimlet also provided estimates of allelic dropout, false alleles and five other specific types of genotyping errors [8,9]. To quantify the power of the microsatellite loci to differentiate individuals, we calculated the theoretical probabilities of identity (the probability that two individuals share the same genotype) for random unrelated individuals (unbiased P_{ID}) and for sibs ($P_{ID\text{-}SIB}$) [10]. $P_{ID\text{-}SIB}$ provides a conservative upper bound for the probability of observing identical multilocus genotypes between two individuals sampled from a population [10]. The multilocus consensus genotypes were compared using the Identity Analysis function in Cervus 3.0.7 [11] to identify resampled individuals and possible mismatches due to genotyping errors. We used the program MM-Dist [12] to compute observed and expected (the latter

for both unrelated individuals and full siblings) distributions of genotypic differences (loci mismatches, k-MM) between samples. We performed an analysis of individual heterozygosity (H-ind) across loci in the final data set, using the H-individual option in GenAlEx 6.502 [13,14] to assess the presence of individuals with unusually low levels of heterozygosity, which may indicate allelic dropout. The final dataset was further tested for scoring and typographical errors, large allele dropout and null alleles using MICRO-CHECKER 2.2.3 [15]. Hardy-Weinberg and linkage equilibria among loci were tested in GENEPOP 4.7.0 [16] using all individuals from the APNR and, taking into account potential non-independence of observations of alleles due to genetic relatedness among individuals and family genetic structure (e.g., Reference [17]), a set of 40 unrelated individuals. The individuals selected were the 40 with the lowest average pairwise relatedness. Pairwise relatedness between all individuals was estimated in ML-RELATE [18]. In the tests for Hardy-Weinberg and linkage equilibria, significance levels (α = 0.05) were adjusted for multiple comparisons using the sequential Holm-Bonferroni procedure [19] in an Excel calculator [20]. Null alleles can bias the estimation of allele frequencies, potentially affecting any subsequent population genetic analyses [21–24]. Determining the presence and frequency of null alleles can be challenging, however, because they may be difficult to distinguish from other genotyping errors, and most methods for estimating null alleles assume panmixia [15,25,26]. Inbreeding and non-null genotyping failure can inflate null allele frequency estimates [25,27]. Studies comparing methods for detection and frequency estimation of null alleles have shown that they have different strengths and limitations, and that combining tests provides greater confidence in the results [28]. Thus, in addition to MICRO-CHECKER and the method of Van Oosterhout et al. [15] to estimate null allele frequencies, we assessed the presence of null alleles and their frequencies using other algorithms: i) an iterative estimator accounting for the presence of null allele homozygotes [26], as implemented in CERVUS; ii) a maximum-likelihood (ML) estimator accounting for both null allele homozygotes and non-null genotyping failure [25], implemented in ML-NULLFREQ; and iii) both an individual inbreeding model (IIM; Bayesian) and a population inbreeding model (PIM; ML) approaches [27], available in INEST 2.2; in the former approach, models including null alleles, inbreeding, genotyping failures, and all combinations of these parameters, were compared using the Deviance Information Criterion (DIC) to select the model that best fit the data.

References

1. Comstock, K.E.; Wasser, S.K.; Ostrander, E.A. Polymorphic microsatellite DNA loci identified in the African elephant (*Loxodonta africana*). *Mol. Ecol.* **2000**, *9*, 1004–1006.
2. Eggert, L.S.; Ramakrishnan, U.; Mundy, N.I.; Woodruff, D.S. Polymorphic microsatellite DNA markers in the African elephant (*Loxondonta africana*) and their use in the Asian elephant (*Elephas maximus*). *Mol. Ecol.* **2000**, *9*, 2223–2225.
3. Archie, E.A.; Moss, C.J.; Alberts, S.C. Characterization of tetranucleotide microsatellite loci in the African Savannah Elephant (*Loxodonta africana africana*). *Mol. Ecol. Notes* **2003**, *3*, 244–246.
4. Brownstein, M.J.; Carpten, J.D.; Smith, J.R. Modulation of non-templated nucleotide addition by Taq DNA polymerase: primer modifications that facilitate genotyping. *BioTechniques* **1996**, *20*, 1004–1010.
5. Frantz, A.C.; Pope, L.C.; Carpenter, P.J.; Roper, T.J.; Wilson, G.J.; Delahay, R.J.; Burke, T. Reliable microsatellite genotyping of the Eurasian badger (*Meles meles*) using faecal DNA. *Mol. Ecol.* **2003**, *12*, 1649–1661.
6. Hansen, H.; Ben-David, M.; Mcdonald, D.B. TECHNICAL ADVANCES: Effects of genotyping protocols on success and errors in identifying individual river otters (*Lontra canadensis*) from their faeces. *Mol. Ecol. Resour.* **2008**, *8*, 282–289.
7. Ahlering, M.A.; Hailer, F.; Roberts, M.T.; Foley, C. A simple and accurate method to sex savannah, forest and asian elephants using noninvasive sampling techniques. *Mol. Ecol. Resour.* **2011**, *11*, 831–834.

8. Valière, N. GIMLET: a computer program for analysing genetic individual identification data. *Mol. Ecol. Resour.* **2002**, *2*, 377–379.
9. Broquet, T.; Petit, E. Quantifying genotyping errors in noninvasive population genetics. *Mol. Ecol.* **2004**, *13*, 3601–3608.
10. Waits, L.P.; Luikart, G.; Taberlet, P. Estimating the probability of identity among genotypes in natural populations: cautions and guidelines. *Mol. Ecol.* **2001**, *10*, 249–256.
11. Kalinowski, S.T.; Taper, M.L.; Marshall, T.C. Revising how the computer program cervus accommodates genotyping error increases success in paternity assignment. *Mol. Ecol.* **2007**, *16*, 1099–1106.
12. Kalinowski, S.T.; Sawaya, M.A.; Taper, M.L. Individual identification and distribution of genotypic differences between individuals. *J. Wildl. Manag.* **2006**, *70*, 1148–1150.
13. Peakall, R.; Smouse, P.E. GenAlEx 6: genetic analysis in Excel. Population genetic software for teaching and research. *Mol. Ecol. Notes* **2006**, *6*, 288–295.
14. Peakall, R.; Smouse, P.E. GenAlEx 6.5: genetic analysis in Excel. Population genetic software for teaching and research--an update. *Bioinformatics* **2012**, *28*, 2537–2539.
15. Van Oosterhout, C.; Hutchinson, W.F.; Wills, D.P.M.; Shipley, P. MICRO-CHECKER: software for identifying and correcting genotyping errors in microsatellite data. *Mol. Ecol. Notes* **2004**, *4*, 535–538.
16. Rousset, F. genepop'007: a complete re-implementation of the genepop software for Windows and Linux. *Mol. Ecol. Resour.* **2008**, *8*, 103–106.
17. Rodríguez-Ramilo, S.T.; Wang, J. The effect of close relatives on unsupervised Bayesian clustering algorithms in population genetic structure analysis. *Mol. Ecol. Resour.* **2012**, *12*, 873–884.
18. Kalinowski, S.T.; Wagner, A.P.; Taper, M.L. ml-relate: a computer program for maximum likelihood estimation of relatedness and relationship. *Mol. Ecol. Notes* **2006**, *6*, 576–579.
19. Holm, S. A simple sequentially rejective multiple test procedure. *Scand. J. Stat.* **1979**, *6*, 65–70.
20. Gaetano, J. Holm-Bonferroni sequential correction: An EXCEL calculator (1.2) [Microsoft Excel workbook]. **2013**.
21. Carlsson, J. Effects of microsatellite null alleles on assignment testing. *J. Hered.* **2008**, *99*, 616–623.
22. Chapuis, M.-P.; Estoup, A. Microsatellite null alleles and estimation of population differentiation. *Mol. Biol. Evol.* **2007**, *24*, 621–631.
23. Dakin, E.E.; Avise, J.C. Microsatellite null alleles in parentage analysis. *Heredity* **2004**, *93*, 504–509.
24. Huang, K.; Ritland, K.; Dunn, D.W.; Qi, X.; Guo, S.; Li, B. Estimating relatedness in the presence of null alleles. *Genetics* **2016**, *202*, 247–260.
25. Kalinowski, S.T.; Taper, M.L. Maximum likelihood estimation of the frequency of null alleles at microsatellite loci. *Conserv. Genet.* **2006**, *7*, 991–995.
26. Summers, K.; Amos, W. Behavioral, ecological, and molecular genetic analyses of reproductive strategies in the Amazonian dart-poison frog, *Dendrobates ventrimaculatus*. *Behav. Ecol.* **1997**, *8*, 260–267.
27. Chybicki, I.J.; Burczyk, J. Simultaneous estimation of null alleles and inbreeding coefficients. *J. Hered.* **2009**, *100*, 106–113.
28. Dąbrowski, M.J.; Bornelöv, S.; Kruczyk, M.; Baltzer, N.; Komorowski, J. 'True' null allele detection in microsatellite loci: a comparison of methods, assessment of difficulties and survey of possible improvements. *Mol. Ecol. Resour.* **2015**, *15*, 477–488.

Supplementary Tables

Supplementary Table 1. Multilocus microsatellite genotypes of the 294 elephants included in this study. For each individual, the Table lists the code number ('ID'), area in which it was sampled, sex, age class ('Age'; adult or juvenile), and the fragment sizes in base pairs of the two alleles at each of the 18 loci (columns 'FH1' to 'LaT25'). Zero alleles represent missing data.

Supplementary Table 2. Hardy-Weinberg equilibrium (HWE) test *p* values from GENEPOP for the entire APNR dataset and for a subset of 40 unrelated individuals.

**Locus**	**HWE *p* value (APNR / 40 unrelated individuals)**
FH1	0.8233 / 0.9547
FH19	0.2993 / 0.8079
FH39	0.5446 / 0.8925
FH40	0.8427 / 0.6035
FH48	0.6845 / 0.9411
FH60	0.9440 / 0.4153
FH67	**0.0024*** / 0.0968
FH71	0.1190 / 0.1230
FH94	0.6824 / 0.2621
FH103	0.1191 / 0.0359*
LA5	0.2678 / 0.0730
LA6	0.3787 / 0.3935
LaT06	**0.0000*** / 0.2869
LaT08	0.3649 / 0.2066
LaT13	0.0072* / 0.1977
LaT18	0.5099 / 0.5723
LaT24	0.4463 / 0.5895
LaT25	**0.0007*** / 0.2919

Asterisks mark *p* values < 0.05, with boldface indicating those that remained significant after sequential Holm-Bonferroni's correction.

Supplementary Table 3. Loci suggested to have null alleles, and their frequencies, as determined by different methods using the APNR dataset. Loci in bold were identified by MICRO-CHECKER. Asterisks mark loci found to be out of Hardy-Weinberg equilibrium by the respective method, and values in italics indicate estimates significantly greater than zero as assessed by a Z-test advised in the manual of INEST for PIM.

	**FH71**	**LaT06**	**LaT13**	**LaT25**	FH67
**Van Oosterhout estimator**	0.06	0.09	0.04	0.08	0.03
**ML-NULLFREQ**	0.04	0.08*	0.03*	0.09*	0.04*
**CERVUS**	0.06	0.10*	0.04	0.09*	0.03
**INEST (IIM)**	0.00	0.08	0.02	0.12	0.03
**INEST (PIM)**	*0.04*	*0.08*	*0.03*	*0.09*	*0.04*

Supplementary Table 4. Main results of the Mantel tests of isolation by distance for all adults and for each sex separately, and for either APNR + KNP or APNR alone. Mantel tests used Nason's kinship coefficient and Rousset's distance as genetic distance measures between individuals, and raw ('Linear') and log-transformed ('Log') pairwise geographic distances.

	**Nason's kinship coefficient**				**Rousset's distance**			
	**Linear**		**Log**		**Linear**		**Log**	
	r_{XY}	*p*	r_{XY}	*p*	r_{XY}	*p*	r_{XY}	*p*
**APNR + KNP adults**	-0.005	**0.038**	-0.004	0.137	0.026	0.258	-0.032	0.202
**APNR + KNP adult females**	0.017	**0.022**	-0.048	**0.000**	-0.014	0.400	0.005	0.470
**APNR + KNP adult males**	0.004	0.115	-0.004	0.245	-0.059	0.131	-0.055	0.129
**APNR adults**	-0.013	**0.001**	-0.020	**0.001**	-0.070	0.054	-0.052	0.073
**APNR adult females**	-0.063	**0.000**	-0.094	**0.000**	0.011	0.443	0.043	0.228
**APNR adult males**	-0.013	**0.016**	-0.020	**0.013**	-0.057	0.124	-0.027	0.278

'r_{XY}' is the correlation coefficient and '*p*' is the *p* value. Significant *p* values are bolded.

Supplementary Table 5. Parameter estimates from the Migraine analyses of APNR, APNR + KNP, and KNP, using either the 'OnePop' model or the 'OnePopVarSize' model.

	**'OnePop' model**			**'OnePopVarSize' model**		
	**APNR**	**APNR + KNP**	**KNP**	**APNR**	**APNR + KNP**	**KNP**
**pGSM**	0.392 [0.303 – 0.474]	0.393 [0.306 – 0.474]	0.358 [0.251 – 0.457]	0.357 [0.262 – 0.447]	0.354 [0.264 – 0.447]	0.308 [0.193 – 0.420]
$\boldsymbol{\Theta}$	2.948 [2.361 – 3.660]	2.890 [2.324 – 3.581]	2.796 [2.144 – 3.635]	2.862 [2.267 – 3.568]	2.813 [2.270 – 3.489]	2.644 [2.004 – 3.472]
$\boldsymbol{N_e}$	737 [590 – 915]	723 [581 – 895]	699 [536 – 909]	716 [567 – 892]	703 [568 – 872]	661 [511 – 868]
	7370 [5900 – 9150]	7230 [5810 – 8950]	6990 [5360 – 9090]	7160 [5670 – 8920]	7030 [5680 – 8720]	6610 [5110 – 8680]
$\boldsymbol{\Theta_{anc}}$	-	-	-	571.9 [1.69 x 10^{-5} – NA]	5290 [1.07 x 10^{-4} – NA]	2734 [7.01 x 10^{-4} – NA]
$\boldsymbol{N_{anc}}$	-	-	-	1.43 x 10^{5} [0.004 – NA]	1.3 x 10^{6} [0.027 – NA]	6.84 x 10^{5} [0.18 – NA]
				1.43 x 10^{6} [0.04 – NA]	1.3 x 10^{7} [0.27 – NA]	6.84 x 10^{6} [1.8 – NA]
$\boldsymbol{N_{ratio}}$	-	-	-	0.005 [4.5 x 10^{-4} – 1.9 x 10^{5}]	5.3 x 10^{-4} [1.13 x 10^{-5} – 28,834]	9.7 x 10^{-4} [4.03 x 10^{-4} – 3,998]
$\boldsymbol{D}$	-	-	-	29.15 [0.032 – 400,954]	41.1 [0.106 – 25,092]	33.46 [1.568 – 57,675]
$\boldsymbol{T}$	-	-	-	495,550 [544 – 6.8 x 10^{9}]	698,700 [1,802 – 4.3 x 10^{8}]	568,820 [26,656 – 9.8 x 10^{8}]
				5 x 10^{6} [5,440– 6.8 x 10^{10}]	7 x 10^{6} [1.8 x 10^{4}– 4.3 x 10^{9}]	5.7 x 10^{6} [2.7 x 10^{5} – 9.8 x 10^{9}]

pGSM: parameter for the geometric distribution of mutation sizes; Θ: mutation-scaled current N_e; N_e: current N_e; Θ_{anc}: mutation-scaled ancestral N_e; N_{anc}: ancestral N_e; $N_{ratio} = \Theta/\Theta_{anc}$; D: scaled age of the demographic change; T: years since the demographic change. The equation $\Theta = 4N_e\mu$, where μ is the mutation rate, was used to convert, respectively, estimates of Θ into estimates of N_e and estimates of Θ_{anc} into estimates of N_{anc}. The equation $T = (D \times G_T)/\mu$, where G_T is the generation time and μ is the mutation rate, was used to convert estimates of D into estimates of T. An average mutation rate of either 10^{-3} or 10^{-4} was assumed, and a generation time of 17 years. Values in parenthesis are 95% confidence intervals.

Supplementary Table 6. VarEff estimates of Ne in the last 3,000 generations for the APNR, assuming TPM mutations at a rate 10^{-3} and two past changes in N_e.

Supplementary Table 7. VarEff estimates of Ne in the last 3,000 generations for APNR + KNP, assuming TPM mutations at a rate 10^{-3} and two past changes in N_e.

Supplementary Table 8. Bottleneck tests for recent reduction in Ne. Deviations from mutation-drift equilibrium heterozygosity were assessed with sign and Wilcoxon's signed rank tests.

Area	Mutation Model	Sign test				Wilcoxon test		
		Expected number of loci with h. e.	Number of loci with h. d.	Number of loci with h. e.	*p*	*p* (1-tail for h. d.)	*p* (1-tail for h. e.)	*p* (2-tails for h. e. and h. d.)
**APNR + KNP**	**I.A.M.**	10.31	2	16	**0.004**	0.999	**0.000**	**0.000**
	**T.P.M. 95% p**	10.63	15	3	**0.000**	**0.001**	0.999	**0.002**
	**T.P.M. 78% p**	10.55	10	8	0.163	0.077	0.929	0.154
	**S.M.M.**	10.64	16	2	**0.000**	**0.000**	0.999	**0.000**
**APNR**	**I.A.M.**	10.26	2	16	**0.004**	0.999	**0.000**	**0.000**
	**T.P.M. 95% p**	10.61	15	3	**0.000**	**0.002**	0.998	**0.003**
	**T.P.M. 78% p**	10.55	10	8	0.163	0.123	0.886	0.246
	**S.M.M.**	10.67	16	2	**0.000**	**0.000**	0.999	**0.000**
**KNP**	**I.A.M.**	10.40	4	14	0.066	0.985	**0.017**	**0.034**
	**T.P.M. 95% p**	10.62	13	5	**0.007**	**0.027**	0.976	0.054
	**T.P.M. 78% p**	10.65	10	8	0.151	0.275	0.739	0.551
	**S.M.M.**	10.71	14	4	**0.001**	**0.004**	0.996	**0.009**

IAM: infinite alleles model; TPM 95%: two-phase model with 95% single-step mutations; TPM 78%: two-phase model with 78% single-step mutations; SMM: stepwise mutation model; h. e.: heterozygosity excess; h. d.: heterozygosity deficiency; *p*: *p* value; 1-tail: one-tailed test; 2-tails: two-tailed test. Significant *p* values are bolded.

Supplementary Table 9. Results of M-ratio tests.

	**APNR** (*M* **= 0.905**)	**APNR + KNP** (*M* = 0.904)	**KNP** (*M* = 0.853)
**$\Theta = 10$; $p_g = 0.10$; $\Delta_g = 3.5$**	$M_c = 0.770$	$M_c = 0.773$	$M_c = 0.723$
**$\Theta = 0.1$; $p_g = 0.10$; $\Delta_g = 3.5$**	$M_c = 0.873$	$M_c = 0.871$	$M_c = 0.871$*
**$\Theta = 10$; $p_g = 0.22$; $\Delta_g = 3.1$**	$M_c = 0.765$	$M_c = 0.768$	$M_c = 0.697$
**$\Theta = 0.1$; $p_g = 0.22$; $\Delta_g = 3.1$**	$M_c = 0.804$	$M_c = 0.802$	$M_c = 0.804$

M: mean *M*-ratio across loci; M_c: critical value for significance of *M*; Θ: mutation-scaled effective population size; p_g: proportion of multi-step mutations; Δ_g: average size of multi-step mutations. Significant tests are indicated by an asterisk next to the critical value.

Supplementary Figures

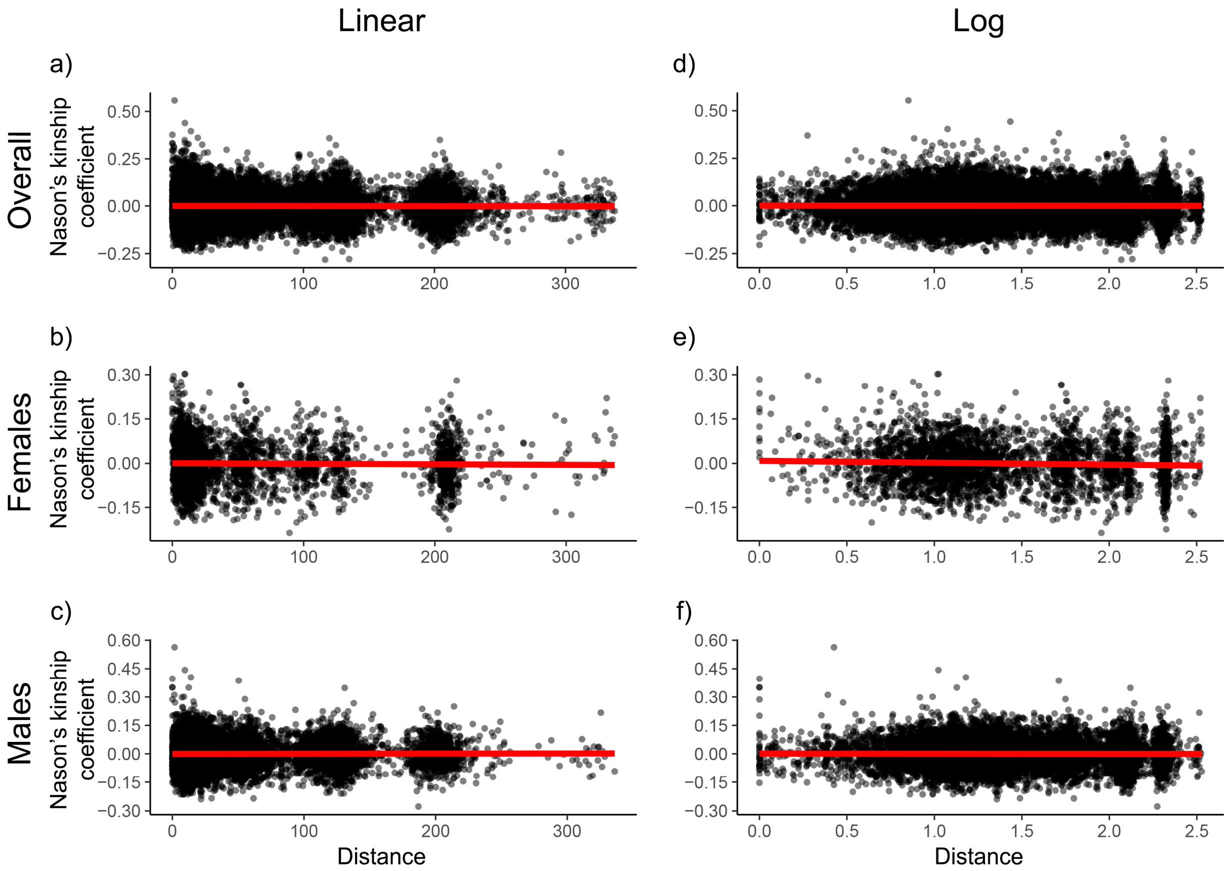

Supplementary Figure 1. Plots of Mantel tests between pairwise genetic and geographic (linear in km and log-transformed) distances for all APNR + KNP adult samples (a and d) and separately for each sex (females: b and e; males: c and f). Dots represent pairwise individual comparisons, and regression lines are in red.

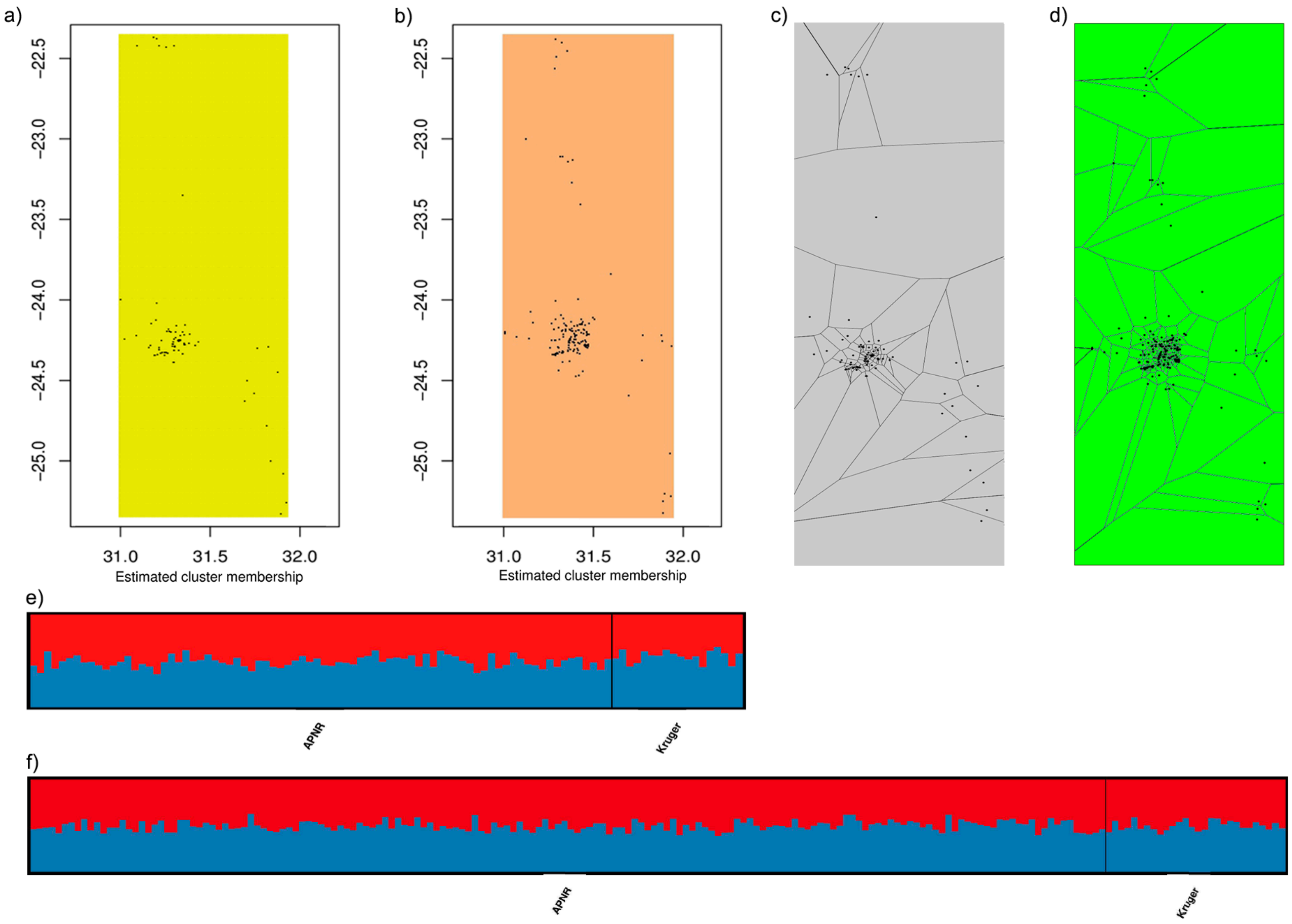

Supplementary Figure 2. Bayesian clustering results from separate analyses by sex of the APNR + KNP samples. a) and b): maps of estimated cluster membership for the most supported $K = 1$ under the uncorrelated allele frequency model in GENELAND for, respectively, females and males; c) and d): Voronoi tessellation maps for the most supported $K = 1$ in the no-admixture model of TESS for, respectively, females and males; e) and f): bar plots from STRUCTURE when $K = 2$ for, respectively, females and males. Each individual is depicted by a column that is partitioned into K segments, which length is proportional to the

membership coefficient of the individual for each cluster. A vertical black line separates individuals from the two areas, which are labeled below the figures. The dots in a), b), c) and d) represent the sampling location of individuals.

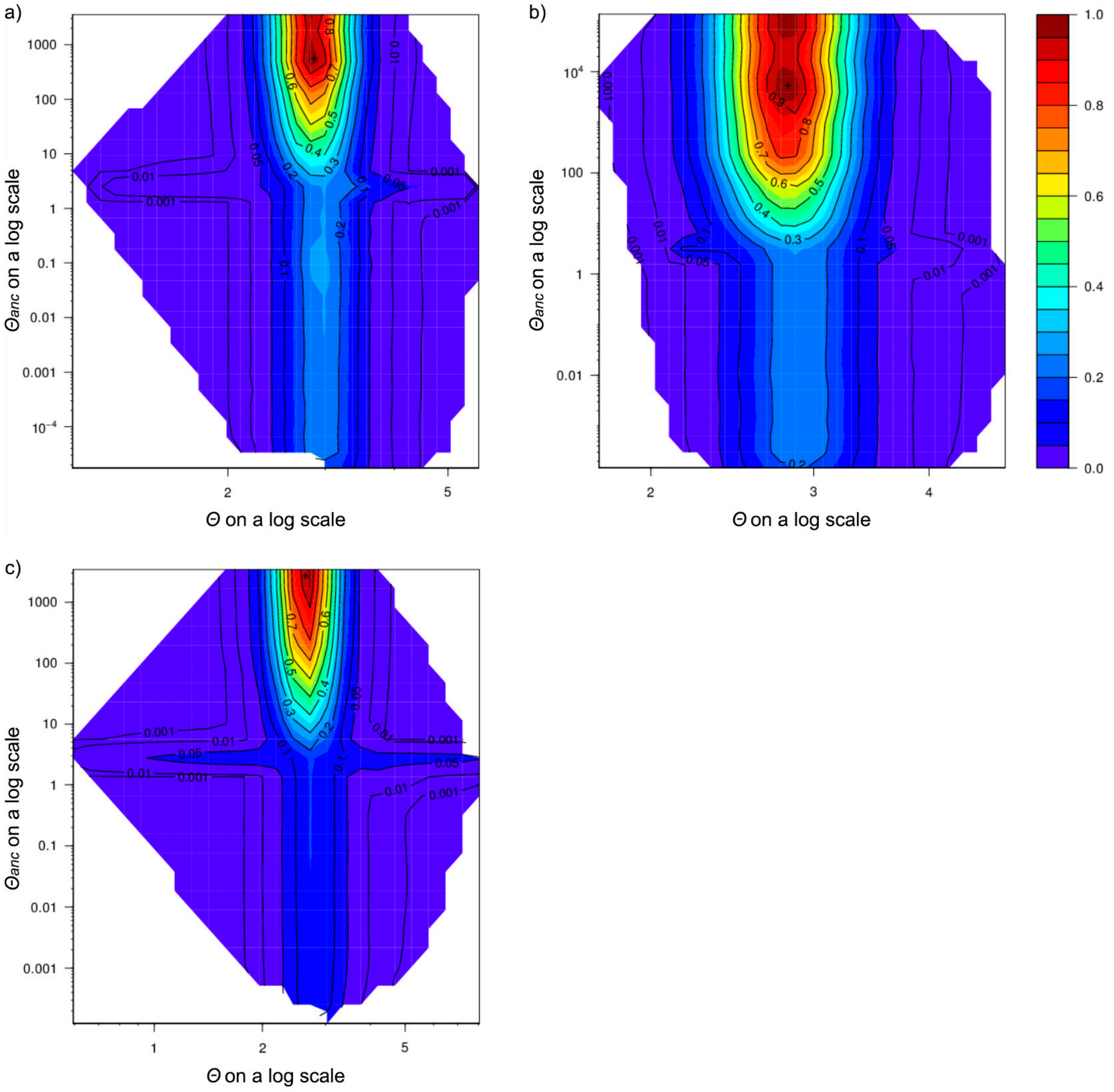

Supplementary Figure 3. Two-dimensional profile likelihood regions of Θ and Θanc from Migraine's 'OnePopVarSize' model for: a) APNR, b) APNR + KNP, and c) KNP. Θ and Θ_{anc}, both in log scale, are in the x and y axes, respectively. The signal for an old bottleneck is not significant. The point estimates and 95% confidence intervals for the two parameters are given in Supplementary Table 5.

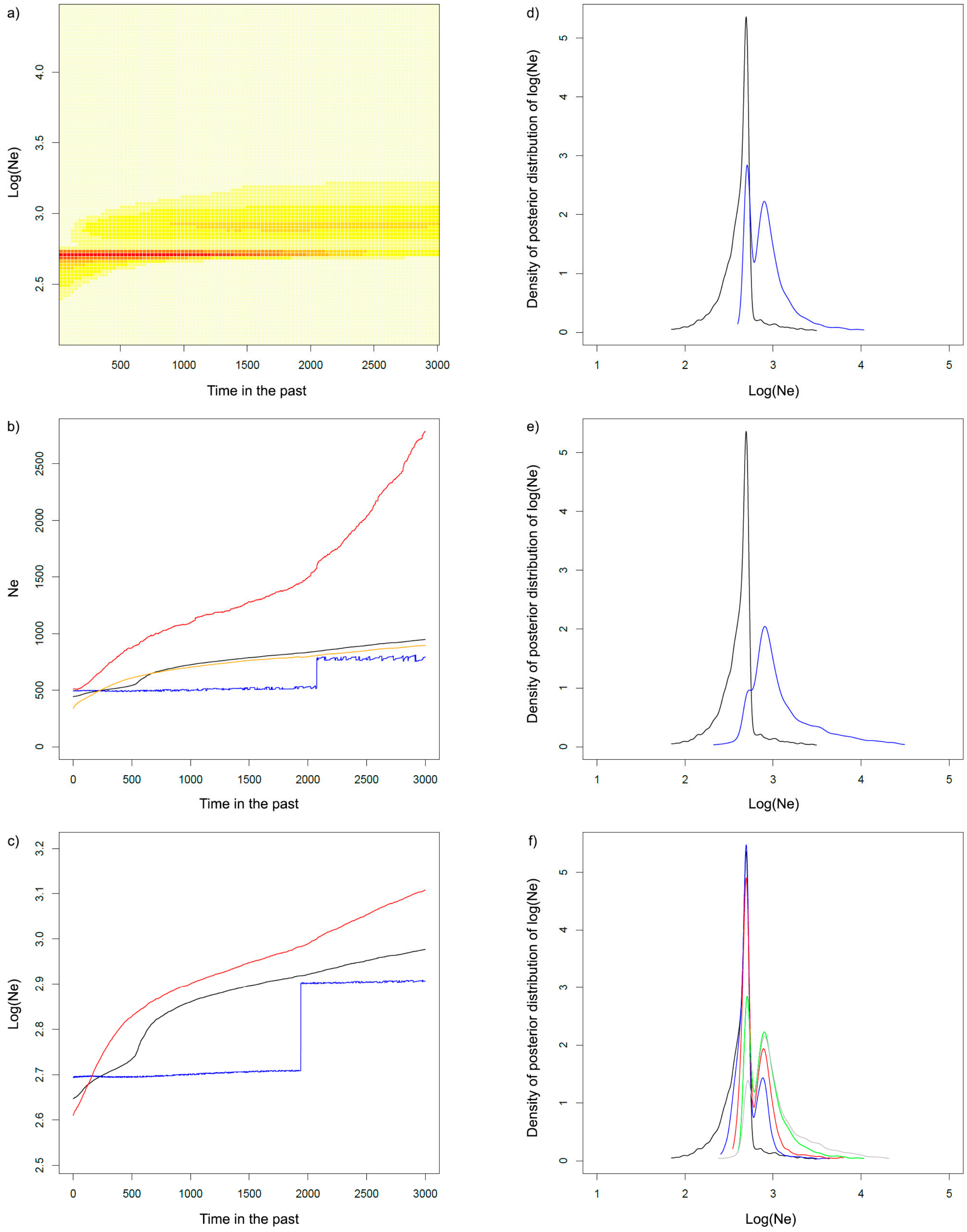

Supplementary Figure 4. VarEff inference of demographic history over the last 3,000 generations based on the APNR + KNP data set. a) Joint posterior distribution for logN_e (*y*-axis) over time in generations (*x*-axis); b) and c) mean (red), median (black), mode (blue) and harmonic mean (orange) of N_e estimates in linear and logarithmic scales, respectively; d), e) and f) densities of the posterior distributions of logN_e for the present and at different times in the past. d) logN_e estimates for the present (black) and 1,500 generations ago (blue), e) logN_e estimates for the present (black) and 3,000 generations ago (blue), and f) logN_e estimates for the present (black) and 500 (blue), 1,500 (red) and 2,500 (green) generations ago.
